# Premature transcription termination modulates stochastic gene expression in bacteria

**DOI:** 10.1126/sciadv.aed0831

**Published:** 2026-05-15

**Authors:** Shafagh Moradian, Nida Ali, Rahul Jagadeesan, Volker Behrends, Andre Sanches Ribeiro, Christoph Engl

**Affiliations:** ^1^ School of Biological and Behavioural Sciences, Queen Mary University of London, London, UK.; ^2^ Laboratory of Biosystems Dynamics, Faculty of Medicine and Health Technology, Tampere University, Tampere, Finland.; ^3^ School of Medicine and Biosciences, University of West London, London, UK.

## Abstract

Bacteria regulate transcription by controlling its initiation and prematurely terminating it through attenuation. Transcriptional regulation produces RNA bursts defined by size and frequency. While bursting via regulated initiation is well characterized, how attenuation shapes burst dynamics remains poorly understood. We show that in *Escherichia coli*, attenuation combined with regulated initiation in the tryptophan operon (*trp*) modulates burst size and frequency, producing switch-like transitions in transcriptional activity between the tryptophan presence and absence. In *Bacillus subtilis*, lacking regulated *trp* initiation, attenuation modulates burst size, gradually changing transcriptional activity as the tryptophan concentration increases. These distinct architectures may reflect adaptive strategies: *B. subtilis* maintains highly expressing cells over a wider tryptophan range, likely beneficial in soil where tryptophan is scarce and unevenly distributed, while *E. coli* enables rapid repression suited to fluctuating gut environments. Notably, *trp* transcription is not strictly cell-autonomous. Highly expressing cells can suppress transcription in neighboring cells, revealing intercellular regulation and supporting community-level cooperation.

## Teaser

Bacteria tune transcription bursts through early termination, enabling switch-like and gradual adaptation to nutrients.

## INTRODUCTION

In both bacteria and eukaryotes, transcriptional output often occurs in bursts, short episodes of mRNA synthesis interrupted by periods of transcriptional silence (*1*–*8*). This transcriptional bursting gives rise to substantial cell-to-cell variability in mRNA levels, even among genetically identical individuals in homogeneous environments (*5*–*10*). Such noise in gene expression facilitates phenotypic heterogeneity, enabling bacterial subpopulations to survive unfavorable conditions such as antibiotic exposure or nutritional depletion (*11*, *12*).

Recent studies have made considerable progress in linking transcriptional bursting dynamics to promoter architecture and transcription initiation mechanisms (*2*, *10*, *13*–*20*). However, postinitiation regulatory mechanisms, such as the premature termination of transcription, remain underexplored in this context, particularly regarding how they influence bursting kinetics at the single-cell level. Addressing this gap is critical to better understand the relationship between gene regulation mechanisms and cellular adaptations. In these conditions, many biosynthetic operons, including those responsible for amino acid production, are subject to premature termination of transcription (*21*, *22*).

**Fig. 1.**
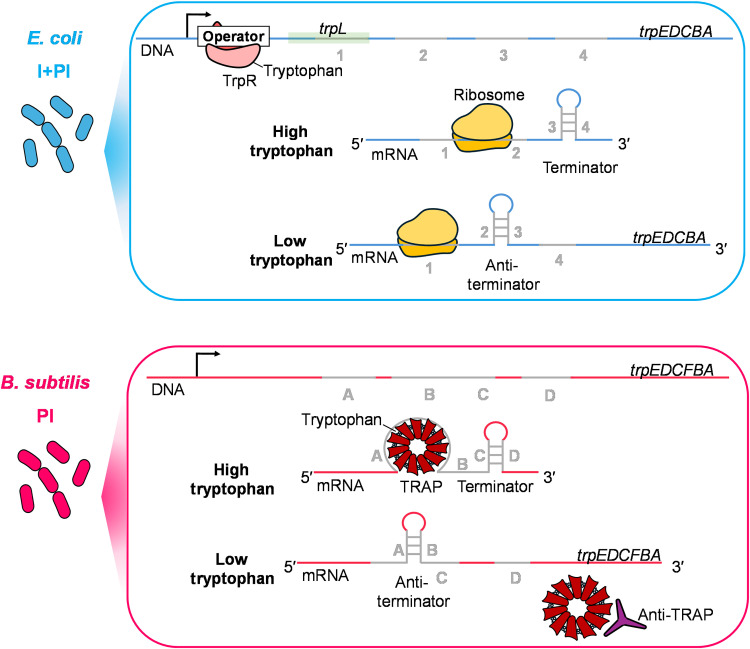
Dual-layer versus single-layer transcription regulation strategies of the *trp* operon. Schematic of the dual-layer (I + PI) regulation in *E. coli* and the PI regulation in *B. subtilis*. In *E. coli*, tryptophan-activated TrpR inhibits transcription initiation by RNA polymerase (RNAP) through binding to the operator region upstream of the structural genes. At the postinitiation level, high levels of charged tRNA^Trp^ promote rapid ribosome translation of *trpL* region 1, which favors the formation of the terminator (3:4) hairpin, leading to transcriptional attenuation. Under tryptophan limitation, TrpR remains inactive, allowing RNAP to initiate transcription, and ribosome stalling at region 1 due to reduced charged tRNA^Trp^ availability enables the formation of the antiterminator hairpin (2:3), resulting in transcriptional read-through of the operon. In *B. subtilis*, RNAP initiates transcription constitutively, and regulation occurs solely at the postinitiation stage. Tryptophan-activated TRAP binds to the leader mRNA to promote terminator hairpin (C:D) formation, which prematurely terminates transcription. Under tryptophan limitation, TRAP activity is reduced because fewer TRAP complexes are loaded with tryptophan and therefore competent for RNA binding. Anti-TRAP further inhibits any residual tryptophan-activated TRAP by preventing its interaction with the leader RNA, thereby allowing the antiterminator (A:B) to form and transcription to proceed into the downstream structural genes of the operon.

A classic model for premature termination of transcription is the tryptophan operon (*trp*), which regulates tryptophan biosynthesis (*23*–*28*). Despite the conservation of the operon across species, *Escherichia coli* and *Bacillus subtilis* have evolved distinct regulatory mechanisms (Fig. 1) (*29*). In *E. coli*, the *trp* operon is regulated by both repression at the level of transcription initiation via the TrpR repressor and attenuation, acting at the postinitiation stage, through the TrpL leader peptide-mediated formation of a transcriptional terminator (*28*, *29*). By contrast, *B. subtilis* primarily relies on postinitiation attenuation mediated by TRAP (*trp* RNA-binding attenuation protein) without a transcriptional regulator acting at initiation (*23*, *30*–*33*). These differing regulatory strategies provide an ideal comparative model to investigate how single-layer [postinitiation only (PI)] versus dual-layer [initiation and postinitiation (I + PI)] control influences transcriptional noise and bursting behavior.

In both organisms, the *trp* operons are transcribed from promoters controlled by housekeeping sigma factors, σ^70^ in *E. coli* and σ^A^ (a member of the σ^70^ family) in *B. subtilis*. In bacteria, σ^70^-dependent promoters drive burst size–modulated transcription when regulated at initiation, producing mRNA in episodic bursts (*2*, *13*). Transcriptional noise in such promoters, however, often decreases at higher expression levels (*9*, *13*, *14*). To date, the effect of premature termination by transcription attenuation on noise and bursting in these promoters remains poorly understood and is the focus of this study (*2*, *13*).

## RESULTS

### Single-layer transcriptional regulation by attenuation allows the persistence of highly expressing subpopulations

To compare the effects of postinitiation-only regulation versus a dual-layered mechanism involving both transcription initiation and postinitiation control on transcriptional activity, we used wild-type (WT) strains of *B. subtilis* and *E. coli*, referred to throughout this article as PI (postinitiation only) and I + PI (initiation and postinitiation) (*34*, *35*). Transcriptional heterogeneity and burstiness were measured across populations exposed to varying concentrations of tryptophan: 0, 5, 50, and 100 μM, denoted as 0, 1×, 10×, and 20×, respectively. This experimental approach enabled us to relate the regulatory architecture to the transcriptional variation across the bacterial population under progressively stronger repression. We used single-molecule RNA (smRNA) fluorescence in situ hybridization (FISH) (*36*) to quantify *trpE* mRNA expression at a single-cell and single-molecule resolution. Representative single-molecule RNA FISH (smRNA FISH) images targeting *trpE* transcripts in *B. subtilis* and *E. coli* are shown in figs. S1 and S2.

**Fig. 2.**
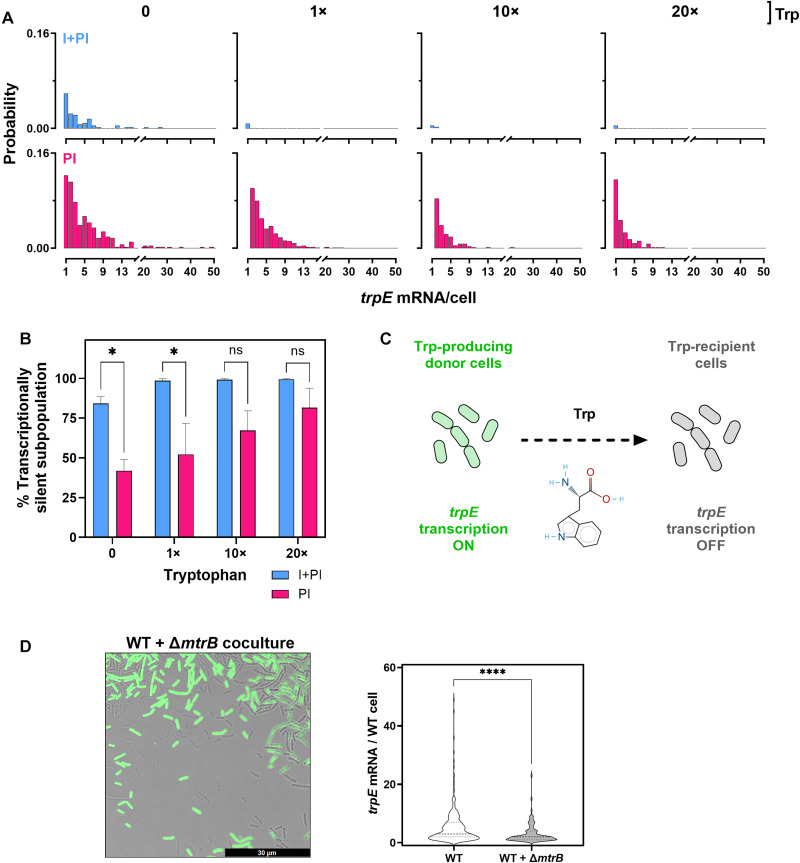
Impact of single- and dual-layer transcriptional regulation on *trpE* transcriptional heterogeneity. I + PI (*E. coli*) and PI (*B. subtilis*) cells were cultured with 0, 1×, 10×, or 20× tryptophan (0, 5, 50, and 100 μM, respectively), and single-cell *trpE* mRNA counts were obtained using smRNA FISH. (**A**) Single-cell mRNA probability distributions of I + PI (top panel) and PI (bottom panel). (**B**) Percentage of transcriptionally silent cells (0 mRNA). Asterisks indicate statistical significance assessed by a two-way analysis of variance (ANOVA) followed by Tukey’s multiple comparisons test. (**C**) Model proposing that tryptophan cross-feeding results in inconsistencies in expression of biosynthetic genes: Active cells synthesize tryptophan and provide it to recipient cells, which then down-regulate *trpE* transcription, creating heterogeneity. (**D**) Left panel: Fluorescence images of WT and GFP-labeled Δ*mtrB* cocultures. *trpE* mRNA molecules were quantified in WT cells grown either alone or with the highly *trpE*-expressing Δ*mtrB* mutant. Scale bar, 30 μM. Right panel: Violin plots showing single-cell *trpE* mRNA distributions in monoculture versus coculture in WT cells with ≥1 *trpE* transcript. The plots demonstrate variability and overall distribution of *trpE* expression. A Mann-Whitney test was performed to assess statistical significance (*P* < 0.0001). Error bars depict standard deviations. ns, not significant. *P < 0.05; ****P < 0.0001.

To assess how regulatory strategies shape transcript output at the single-cell level, we analyzed the single-cell *trpE* mRNA probability distributions (Fig. 2A). The mean and standard deviation of *trpE* mRNA per cell in each condition and strain are provided in table S2. Under tryptophan starvation, both modes showed heterogeneous expression, but the maximum output differed: I + PI cells produced up to 27 transcripts, while PI reached 49. This suggests that the additional layer of repression at transcription initiation in the I + PI system not only reduces the proportion of transcriptionally active cells but also constrains their transcriptional output capacity. With added tryptophan, I + PI cells maintained only one or two transcripts, eliminating high expressors from the population, whereas PI retained a subpopulation of cells with more than five transcripts even at 20× tryptophan, indicating incomplete silencing despite strong attenuation signals. These preadapted cells, defined here as cells that maintain elevated *trpE* transcript levels under tryptophan-rich conditions, may provide a survival advantage at the population level during depletion of environmental tryptophan by creating a reservoir of cells able to rapidly resume biosynthesis when tryptophan availability collapses.

Cells were characterized as transcriptionally silent if they did not contain any *trpE* mRNA molecules. Even in the absence of any exogenously supplemented tryptophan, only a subset of cells exhibited *trpE* expression (Fig. 2B). This persistence of a transcriptionally silent subpopulation under tryptophan-starved conditions was observed in both regulatory modes, but the proportion of inactive cells was double in the dual-layered regulatory system (I + PI), where 84% of cells were inactive, compared to 42% in the single-layer PI model.

Statistical analysis confirmed that the differences in the percentage of transcriptionally inactive cells between the I + PI and PI regulation modes were significant under both 0 and 1× tryptophan conditions (*P* = 0.026 and *P* = 0.019, respectively). As extracellular tryptophan concentrations increased, both modes exhibited an increase in the proportion of transcriptionally inactive subpopulation. However, the magnitude and sensitivity of this response are more pronounced in the I + PI mode, indicating that dual-layered regulation provides a greater capacity to shift transcriptional activity across the population in response to metabolic cues. In contrast, the PI system showed a more gradual, dose-dependent increase in transcriptional inactivation.

### Highly *trpE*-expressing cells suppress *trpE* transcription in recipient cells

The persistence of *trpE*-nontranscribing cells during tryptophan starvation, shown in Fig. 2B, is unexpected, given the amino acid’s essential role in growth. One explanation is the uptake of tryptophan produced by neighboring *trpE*-active cells. We therefore asked whether high *trpE* expressors [here labeled with green fluorescent protein (GFP) to discriminate from WT cells] could supply sufficient tryptophan to suppress transcription in recipient WT cells, allowing a silent subpopulation to persist even without external tryptophan (Fig. 2C).

First, we asked whether tryptophan could be released by *trpE*-expressing producer cells and taken up by auxotrophs lacking *trpE* (fig. S3). Extracellular amino acids were measured in *E. coli* and *B. subtilis* WT cultures grown in minimal media, revealing the presence of tryptophan in supernatants of both species (table S1). This is unlikely to result from cell lysis: Intracellular tryptophan in *E. coli* is <1% of aspartate, yet abundant intracellular amino acids such as aspartate, lysine, and arginine were absent from the supernatant, indicating selective metabolite release. Other costly amino acids, including phenylalanine and tyrosine, were also detected, suggesting cross-feeding of metabolically expensive compounds. The growth of Δ*trpE* auxotrophs on WT spent medium, but not fresh minimal medium, confirmed that tryptophan was released and available for uptake (fig. S4). Together, these data demonstrate that *trpE*-positive producer cells can release tryptophan into the extracellular environment and it can subsequently support the growth of *trpE*-negative nonproducer cells, suggesting the possibility of cross-feeding.

Notably, our data further suggest that cross-feeding can act as a regulatory mechanism of gene expression, enabling transcriptional cross-talk between cells. Using *B. subtilis* coculture as a model, we found that the presence of highly *trpE*-expressing Δ*mtrB* cells (lacking transcription attenuation protein TRAP) suppresses *trpE* transcription at the native chromosomal locus of WT cells (Fig. 2D). Single-cell analysis revealed a 46% decrease in mean *trpE* mRNAs per WT cell in coculture with Δ*mtrB*. The fraction of transcriptionally silent cells increased by 12%, while highly (>5 mRNAs) and ultrahighly (>30 mRNAs) expressing WT cells were reduced or eliminated (fig. S5). This suppression in WT cells likely reflects enhanced tryptophan supply by Δ*mtrB* cells, which triggers TRAP-mediated attenuation of *trpE* transcription in WT cells. Together, these findings show that cross-feeding does not merely support auxotrophic growth but can directly alter transcriptional states within a population, providing a previously unrecognized source of cell-to-cell variation through metabolite-driven regulatory interactions between cells.

### Dual-layer transcriptional regulation drives abrupt shifts in noise and bursting

**Fig. 3.**
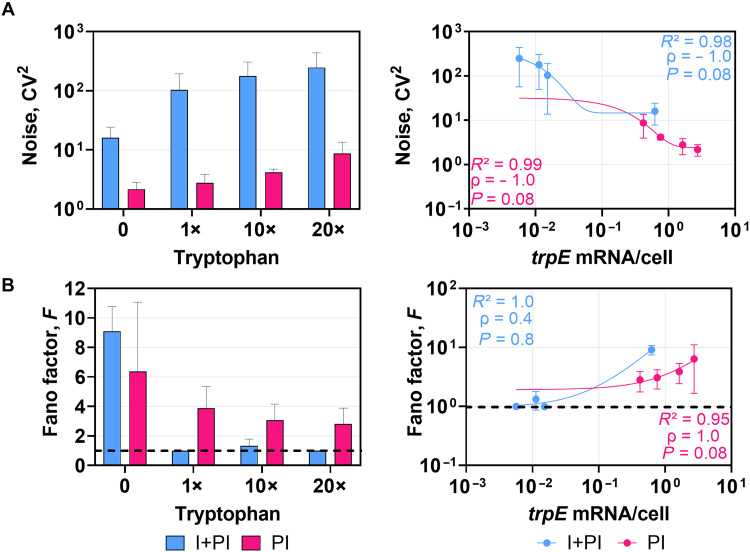
Comparison of noise and bursting of single- and dual-layer transcriptional regulation. (**A**) Noise (*CV*^2^ = σ^2^/μ^2^, where σ represents the standard deviation of the measured mRNA levels, and μ is the mean) and (**B**) burstiness (Fano factor *F* = σ^2^/μ) of *trpE* transcription under each tryptophan concentration (left) and as a function of the mean *trpE* mRNAs per cell (right). *CV*^2^ and *F* were calculated from the mean and standard deviations associated with the mRNA expression levels acquired from the data output from smRNA FISH. The line at Fano = 1 distinguishes bursty event patterns (values above the line) from nonbursty patterns (values below the line). Nonlinear one-phase exponential decay regression was applied to the noise and burstiness versus mean mRNA, and corresponding *R*^2^ values are displayed. Spearman’s rank correlation coefficient (ρ) and the associated *P* values are also shown. Error bars in (A) and (B) depict standard deviations.

To assess how postinitiation mechanisms combined with repression at initiation shape transcriptional noise, we quantified *trpE* transcriptional noise in the I + PI (dual-layer) and PI (single-layer) modes. Tryptophan supplementation increased noise in both modes. Notably I + PI consistently showed higher noise across all concentrations, including 0 tryptophan, indicating that dual regulation inherently promotes greater noise in gene expression regardless of environmental tryptophan availability (Fig. 3A). In PI, noise increased gradually with tryptophan concentrations, whereas in I + PI, noise remained relatively high across the same range.

Noise in *trpE* mRNA expression was plotted as a function of mean expression level and analyzed using nonlinear regression. The relationship was strong, with *R*^2^ values exceeding 0.9 for both I + PI and PI. Spearman’s analysis showed a strong negative correlation between mean *trpE* mRNA levels per cell and noise (ρ = −1.0, *P* = 0.08). In both systems, noise declined as transcription increased, but the pattern differed: PI showed a gradual reduction, whereas I + PI displayed a sharper, more abrupt drop.

Further analysis revealed Fano factors >1 at 0 tryptophan in both modes of regulation, consistent with bursty transcription (Fig. 3B). In PI, burstiness declined gradually but persisted above 1 across all tryptophan levels. In I + PI, however, burstiness dropped sharply with supplementation, approaching Poisson-like values (~1), reflecting a switch to more uniform expression. *B. subtilis*, which lacks initiation control, also exhibited Fano factor >1, showing that burstiness originates downstream from TRAP-mediated attenuation rather than promoter activity.

Burstiness in *trpE* mRNA expression showed a strong nonlinear relationship with mean expression level (*R*^2^ > 0.9 for both I + PI and PI conditions). However, correlation patterns differed: PI showed a strong monotonic relationship (ρ = 1, *P* = 0.08), consistent with gradual expression-dependent reduction in burstiness, whereas I + PI displayed a weaker monotonic trend (ρ = 0.4, *P* = 0.8) because of a plateau followed by an abrupt switch-like increase. Together, these results indicate that PI regulation produces a graded noise-burst response, while dual-layer I + PI regulation enforces an abrupt, switch-like transcriptional response to the tryptophan presence.

### Deconstruction of the PI regulation system

To determine the molecular regulator of transcriptional noise and bursting in *B. subtilis* seen in Fig. 3, we examined two central regulators: TRAP and its inhibitor anti-TRAP (*31*, *32*, *37*, *38*). Raw smRNA FISH images show a marked increase in tetramethylrhodamine (TAMRA) signal in the absence of TRAP (Δ*mtrB*) compared with the absence of anti-TRAP (Δ*rtpA*), consistent with constitutive *trpE* expression at initiation and loss of postinitiation attenuation (fig. S1).

**Fig. 4.**
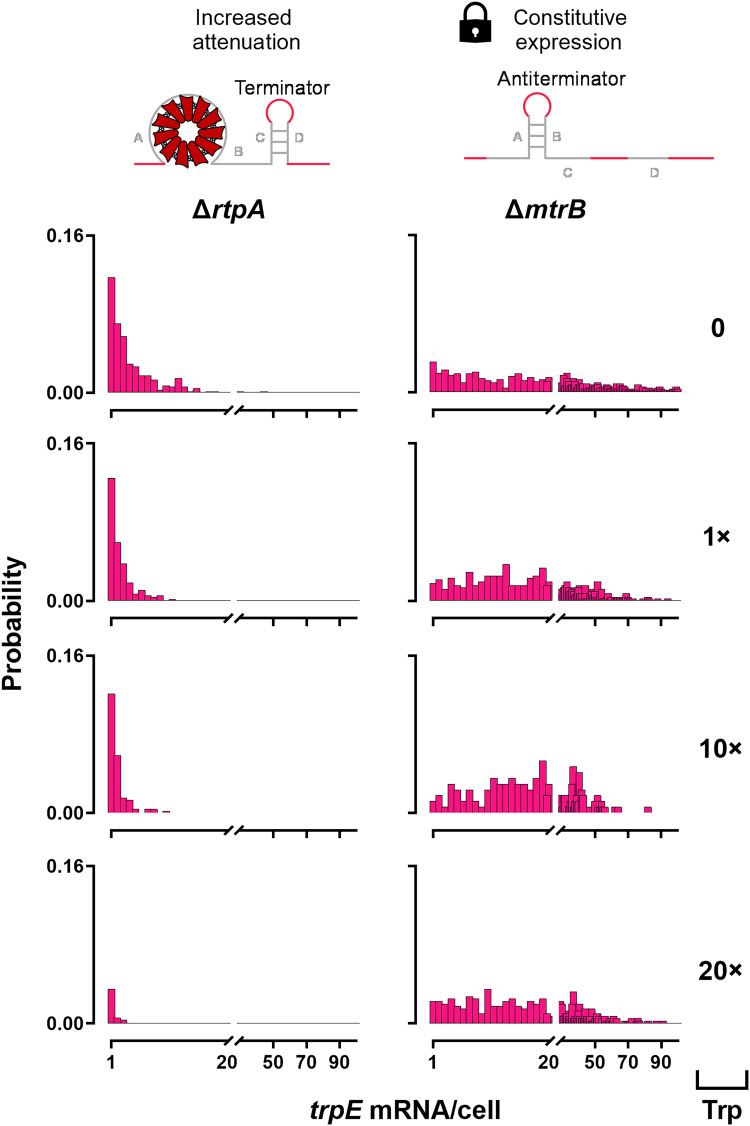
*trpE* mRNA distributions among regulatory mutants of the PI regulation system. Probability distributions of *trpE* mRNA per cell in *B. subtilis* 168 *trpC^+^* lacking anti-TRAP (Δ*rtpA*; left-hand panel) and TRAP (Δ*mtrB*; right-hand panel) in the presence of 0, 1×, 10×, or 20× (0, 5, 50, or 100 μM, respectively) extracellular tryptophan.

Figure 4 illustrates the effects of anti-TRAP and TRAP on the probability distributions of cells expressing one or more *trpE* mRNA. In Δ*rtpA* and, thus, in the absence of anti-TRAP, increasing tryptophan concentrations shift the distribution toward lower mRNA copy numbers. The probability of observing a single *trpE* mRNA molecule per cell remained similar between 0 and 10× tryptophan; however, at 20× tryptophan, this probability is markedly reduced. Consistently, 20× tryptophan also results in a pronounced decrease in the probability of cells containing more than one *trpE* mRNA molecule. This effect at 20× tryptophan was less pronounced in WT cells (Fig. 2A). By contrast, Δ*mtrB* cells lacking TRAP showed a broad expression range of 1 to 100 *trpE* mRNAs per cell under all conditions, indicating persistent read-through in the absence of attenuation (Fig. 4). Notably, tryptophan addition reduced the very highly expressing fraction (>100 mRNA molecules; fig. S7), indicating that tryptophan imposes an upper limit on extreme *trpE* expression even when TRAP is absent.

**Fig. 5.**
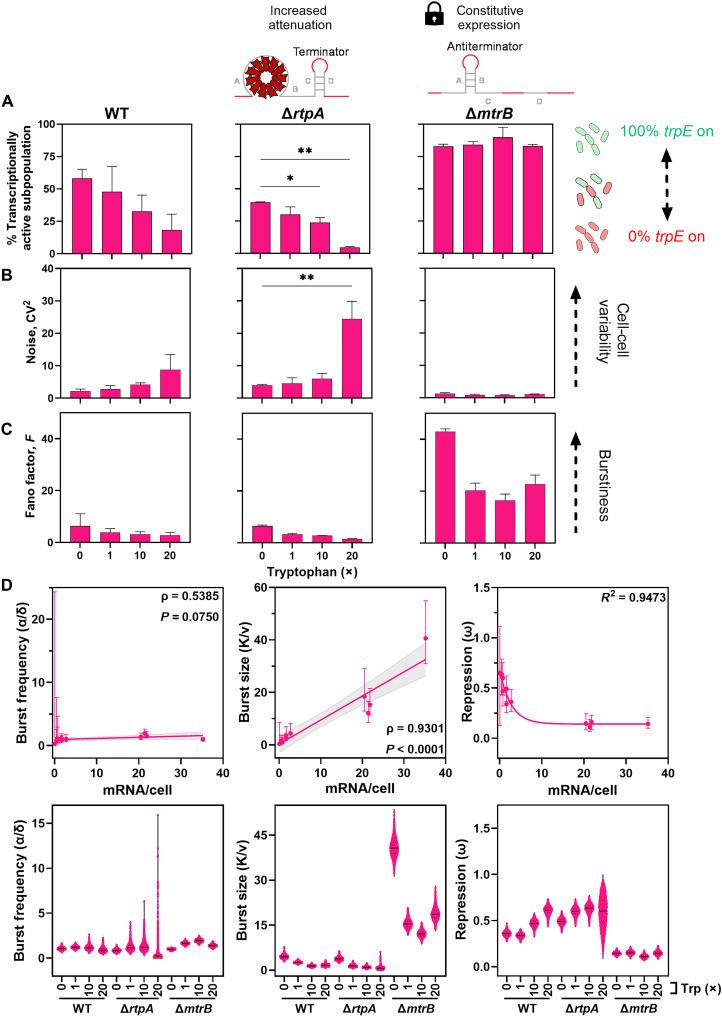
Transcriptional heterogeneity and bursting of regulation by attenuation only. Transcriptional heterogeneity was assessed in strains with either enhancement (Δ*rtpA*) or loss of TRAP-mediated attenuation (Δ*mtrB*) compared to the WT strain. The padlock symbol denotes a genetic “lock” state, in which Δ*mtrB* cells are transcriptionally fixed in the on-state because of compromised attenuation regulation. Cells cultured with 0, 1×, 10×, or 20× tryptophan (0, 5, 50, and 100 μM, respectively). Transcription of *trpE* was quantified using smRNA FISH. (**A**) Percentage of cells in the transcriptionally on state (defined as cells containing ≥1 *trpE* mRNA molecules). (**B**) Transcriptional noise (*CV*^2^ = σ^2^/μ^2^) and (**C**) Fano factor (*F* = σ^2^/μ) calculated from the mean (μ) and standard deviation (σ) of *trpE* mRNA copy number per cell. Mean values ± standard deviations are shown for two independent experiments. Statistical analysis was performed using a one-way ANOVA followed by Dunnett’s multiple comparisons test. (**D**) Burst kinetics of *trpE* transcription in *B. subtilis* strains WT, Δ*rtpA*, and Δ*mtrB* as a function of the mean *trpE* mRNAs per cell (top) and under each tryptophan condition tested (bottom). The error bars represent 95% credible intervals derived from the MCMC posterior distribution. *P < 0.05; **P < 0.01.

Figure 5 shows the effect of TRAP and anti-TRAP on transcriptional heterogeneity and bursting dynamics. The mRNA distributions were fitted with a zero-inflated negative binomial model to extract burst parameters, as described previously (*13*). Overlay comparisons between the data and model fits are shown in fig. S6.

In Δ*rtpA*, the fraction of transcriptionally active cells declined with increasing tryptophan, similar to WT, but significance was only reached at higher concentrations (10× and 20×) (Fig. 5A). This suggests that anti-TRAP sustains a subpopulation of active cells under tryptophan-rich conditions, maintaining heterogeneity. By contrast, Δ*mtrB* cells lacking TRAP showed >80% active cells across all conditions (Fig. 5A), indicating that TRAP is required to establish and maintain a transcriptionally silent subpopulation in response to tryptophan. Noise patterns (Fig. 5B) further supported this role. WT and Δ*rtpA* both showed increasing noise with tryptophan, implicating TRAP as a driver of cell-to-cell variability (Fig. 5B). However, Δ*rtpA* displayed higher noise than WT only at 20× tryptophan, consistent with anti-TRAP acting as a buffer against excessive TRAP-mediated repression under high tryptophan (Fig. 5B). In Δ*mtrB*, noise was consistently low, confirming that TRAP is essential for introducing heterogeneity (Fig. 5B).

Bursting behavior was broadly similar between WT and Δ*rtpA*, with moderate decreases in Fano factor at higher tryptophan and values always >1, confirming bursty transcription (Fig. 5C). By contrast, Δ*mtrB* exhibited higher burstiness than WT and Δ*rtpA* across all tryptophan concentrations; however, the magnitude of burstiness varied across conditions. Burstiness was the highest in the absence of tryptophan and decreased to intermediate levels at 1×, 10×, and 20× tryptophan (Fig. 5C). Thus, TRAP suppresses bursts, while anti-TRAP exerts minimal effects on burst dynamics. We next used mathematical modeling (*13*) to dissect bursting parameters (Fig. 5D). In *B. subtilis*, repression (ω) was similarly low in the absence and presence of low levels of tryptophan (1×) and strongly inversely correlated with mean *trpE* transcription (*R*^2^ > 0.9) as tryptophan levels increased further. In strains without anti-TRAP (Δ*rtpA*), repression increased already to maximum levels at low tryptophan (1×). In addition, anti-TRAP appears to modulate the sensitivity to repression at the transition from no to low levels of tryptophan as Δ*rtpA* showed higher repression even in the absence of tryptophan, comparable to WT at 10× tryptophan. This indicates that anti-TRAP is required to prevent premature attenuation when tryptophan is scarce. At 20× tryptophan, Δ*rtpA* repression was most heterogeneous, consistent with its elevated noise profile (Fig. 5B). Thus, anti-TRAP ensures a more homogeneous transcriptional response to high tryptophan levels. In Δ*mtrB*, repression remained low regardless of tryptophan, confirming TRAP as the main factor mediating transcriptional silencing of the *trp* operon in *B. subtilis*.

Burst size (Fig. 5D) positively correlated with mean expression (ρ = 0.93, *P* < 0.0001). WT and Δ*rtpA* showed modest tryptophan-dependent reductions, but removal of TRAP displayed a substantial increase, demonstrating that TRAP negatively regulates burst size. Burst frequency (Fig. 5D) correlated weakly and nonsignificantly with expression (ρ = 0.54, *P* = 0.08), suggesting the lack of frequency regulation. TRAP deletion had a minimal effect on frequency, although a small Δ*rtpA* subpopulation at 20× tryptophan showed high-frequency bursting, hinting that anti-TRAP may suppress rare cells with high transcriptional frequency states (Fig. 5D). Overall, TRAP-mediated attenuation primarily shapes bursting through the control of burst size (Fig. 5D). Together, these findings show that TRAP is essential for generating transcriptional heterogeneity and bursts by driving repression and limiting burst size, while anti-TRAP fine-tunes system sensitivity and buffers noise under high tryptophan.

### Deconstruction of the I + PI regulation system

We analyzed *E. coli* regulatory mutants to dissect the contributions of transcriptional initiation (TrpR) and postinitiation regulation (attenuation). To isolate the contribution of ribosome-mediated attenuation, we used a Δ*trpR* strain lacking the TrpR repressor. In this strain, transcription is constitutively expressed at the point of initiation, and regulation of transcriptional output occurs through attenuation. To assess the effect of enhanced attenuation in the presence of regulation by TrpR, we used a Δ*trpL* strain lacking the leader sequence necessary for ribosome stalling. In this strain, the absence of stalling leads to constitutive formation of the terminator hairpin, thereby genetically locking the system into a state of maximal attenuation. A double mutant (Δ*trpR*Δ*trpL*) lacking both TrpR and the leader sequence was used to investigate the effect of maximal attenuation in the absence of transcriptional repression at initiation. Although a recent study has identified small RNAs that interact with the *trpL* 5′ untranslated region in *E. coli* and may influence *trpE* expression (*39*), our primary emphasis is on repression occurring at the initiation stage by TrpR and ribosome-mediated attenuation.

**Fig. 6.**
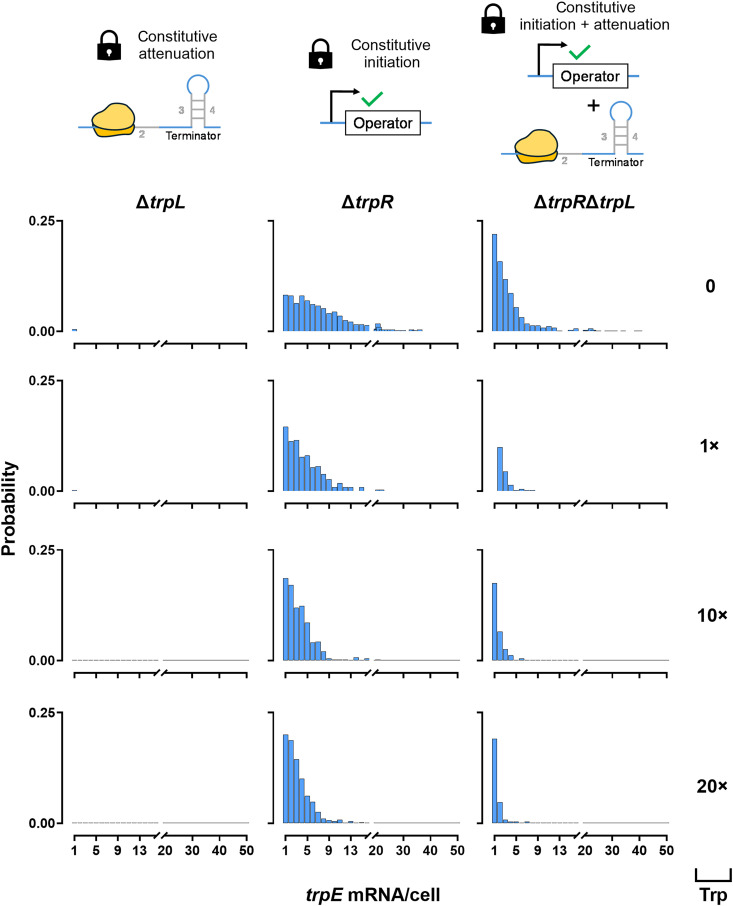
*trpE* mRNA distributions among regulatory mutants of the I + PI regulation system. Probability distributions of *trpE* mRNA per cell in *E. coli* BW25113 lacking *trpL* (Δ*trpL*; left-hand panel), *trpR* (Δ*trpR*; middle panel), or both *trpR* and *trpL* (Δ*trpR*Δ*trpL*; right-hand panel) in the presence of 0, 1×, 10×, or 20× (0, 5, 50, or 100 μM, respectively) extracellular tryptophan.

Raw smRNA FISH images (fig. S7) showed a marked increase in TAMRA signal in Δ*trpR*, reflecting constitutive initiation of *trpE* transcription. In the absence of TrpR (Δ*trpR*), the *E. coli* cells exhibited a substantially broader probability distribution of *trpE* mRNA per cell than WT (compare Figs. 2 and 6). Increasing tryptophan concentrations shifted this distribution toward lower mRNA counts. At 10× and 20× tryptophan, the distribution closely resembled that of the Δ*trpR*Δ*trpL* mutant at 0 tryptophan, indicating that attenuation alone strongly constrains mRNA output (Fig. 6). In Δ*trpR*Δ*trpL,* where TrpR-dependent repression of transcription initiation is absent, transcripts escaped attenuation created by the removal of *trpL*, indicating leaky expression; however, increasing tryptophan supplementation markedly suppressed highly expressing cells, demonstrating that tryptophan sensitivity persists even without TrpR and *trpL*. In Δ*trpL,* by contrast, the probability of detecting any *trpE* mRNA was zero at 10× and 20× tryptophan and very low at 0 and 1× tryptophan, indicating near-complete transcriptional suppression under high tryptophan conditions, demonstrating the strong repressive effect of attenuation and TrpR-dependent control.

**Fig. 7.**
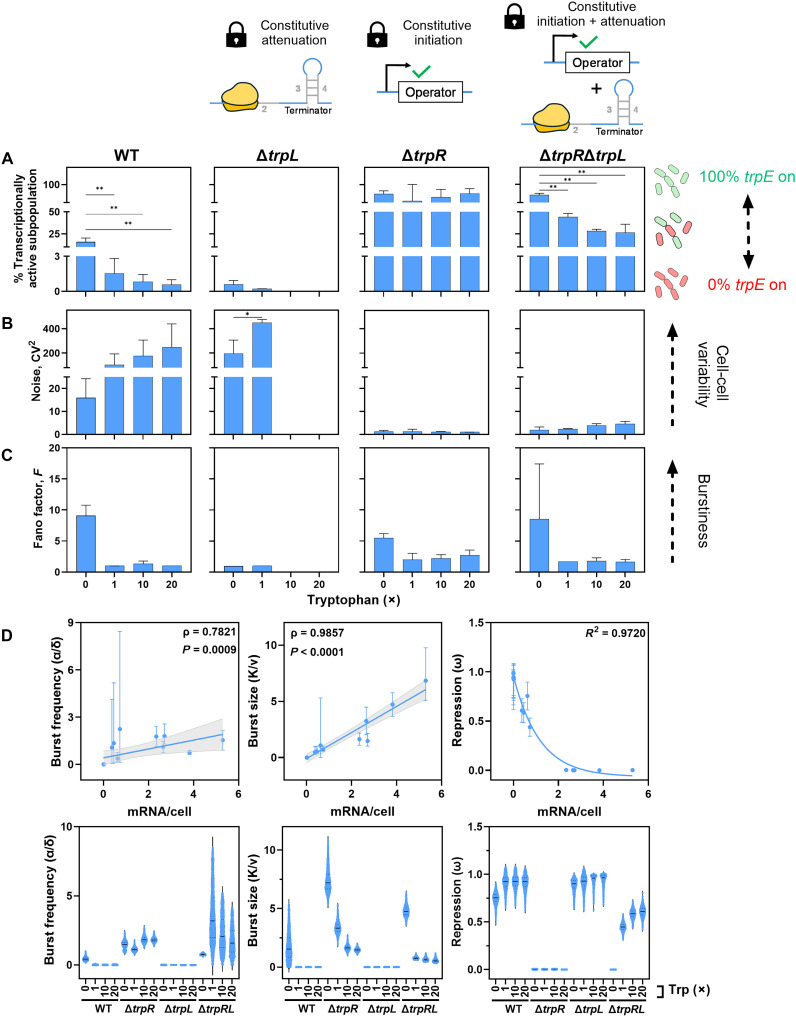
Transcriptional heterogeneity and bursting of combined regulation by repression and attenuation. Transcriptional heterogeneity was assessed in strains with enhanced ribosome-mediated attenuation (Δ*trpL*), constitutive initiation (Δ*trpR*), or both (Δ*trpR*Δ*trpL*) compared to the WT strain with functional initiation and postinitiation regulation. The padlock symbol denotes a genetic “lock” state, in which cells are either genetically fixed in the on-state for transcription attenuation (Δ*trpL*), initiation (Δ*trpR*), or both (Δ*trpR*Δ*trpL*). Cells cultured with 0, 1×, 10×, or 20× tryptophan (0, 5, 50, and 100 μM, respectively). Transcription of *trpE* was quantified using smRNA FISH. (**A**) Percentage of cells in the transcriptionally on state (defined as cells containing ≥1 *trpE* mRNA molecules). (**B**) Transcriptional noise (*CV*^2^ = σ^2^/μ^2^) and (**C**) Fano factor (*F* = σ^2^/μ) calculated from the mean (μ) and standard deviation (σ) of *trpE* mRNA copy number per cell. Mean values ± standard deviations are shown for two independent experiments. Statistical analysis was performed using a one-way ANOVA followed by Dunnett’s multiple comparisons test. (**D**) Burst kinetics of *trpE* transcription in *E. coli* strains WT, Δ*trpL*, Δ*trpR*, and Δ*trpR*Δ*trpL* as a function of the mean *trpE* mRNAs per cell (top) and under each tryptophan condition tested (bottom). The error bars represent 95% credible intervals derived from the MCMC posterior distribution. *P < 0.05; **P < 0.01.

Analysis of transcriptional activity (Fig. 7A) further underscored these differences. In Δ*trpR*, ~70% of cells remained transcriptionally active across all tryptophan concentrations, showing that attenuation alone cannot silence large subpopulations. Δ*trpL* showed decreasing transcriptional activity with tryptophan, reaching full repression at ≥10× tryptophan. This behavior corresponds to enhanced attenuation, together with increased TrpR activity at higher tryptophan concentrations. Consistently, the Δ*trpR*Δ*trpL* double mutant regained transcription at the same tryptophan concentrations (≥10×), confirming that initiation-level repression is responsible for the strong repression in Δ*trpL*. The Δ*trpR*Δ*trpL* mutant resembled Δ*trpR* in the absence of tryptophan (~79% active) but declined with tryptophan supplementation (Fig. 7A). Thus, TrpR primarily dictates population transcriptional activity, with influence by attenuation only when maximized.

Analysis of transcriptional noise (Fig. 7B) revealed that Δ*trpL* exhibited elevated noise without and at low levels of tryptophan compared to WT, peaking at 1×, but dropped to zero when fully repressed (10× to 20×) (fig. S7 and table S2). The Δ*trpR* mutant maintained low transcriptional noise regardless of tryptophan, while Δ*trpR*Δ*trpL* showed a moderate increase in noise at high tryptophan (Fig. 7B). Strains lacking TrpR consistently had lower noise, implicating TrpR as the main source of variability, with limited impact from genetically maximized attenuation (Fig. 7B).

Bursting analysis (Fig. 7C) showed that *trpE* transcription in Δ*trpL* is nonbursty at 0 and 1× tryptophan. By contrast, *trpE* transcription in Δ*trpR* and Δ*trpR*Δ*trpL* remained bursty (Fano factor >1) under all conditions, indicating that the absence of TrpR restores bursty transcription even with maximal attenuation.

Repression (Fig. 7D) inversely correlated with mean *trpE* transcription (*R*^2^ > 0.9). WT and Δ*trpL* showed similarly high repression with tryptophan. However, in the absence of tryptophan, repression was higher in Δ*trpL* than in WT, consistent with attenuation being relieved only under tryptophan starvation (*28*). Cells lacking TrpR (Δ*trpR*) showed minimal repression independent of tryptophan, while additional removal of *trpL* (Δ*trpR*Δ*trpL*) enabled tryptophan-dependent repression (Fig. 7D).

Burst size (Fig. 7D) strongly correlated with mean transcription (ρ = 0.99, *P* < 0.0001). It was higher in the absence than in the presence of TrpR and declined with tryptophan supplementation. However, while, in WT cells, burst size was switched off already at low levels of tryptophan, burst size gradually decreased with increasing tryptophan concentrations in Δ*trpR* cells (Fig. 7D). Removal of *trpL* reduced the burst size compared to its isogenic parent, yet the overall size of bursts was again dependent on the presence or absence of TrpR. If TrpR was present, removal of *trpL* (Δ*trpL*) only had an effect in the absence of tryptophan. Yet, in the absence of TrpR, the removal of *trpL* (Δ*trpR*Δ*trpL*) reduced the burst size compared to Δ*trpR* under all conditions, with a switch-like behavior between the presence and absence of tryptophan. Overall, our results indicate that complete repression of burst size requires regulation at initiation and attenuation (Fig. 7D).

Burst frequency (Fig. 7D) also correlated with expression (ρ = 0.78, *P* = 0.0009), and in WT, it was switched off in the presence of tryptophan. After the removal of TrpR (Δ*trpR*), burst frequency was increased and no longer responsive to tryptophan. Lack of *trpL* in the presence of intact TrpR (Δ*trpL*) prevented burst frequency control under all conditions. Additional lack of *trpL* (Δ*trpR*Δ*trpL*) had no major effect on the elevated median burst frequency observed in (Δ*trpR*); however, it altered the range of burst frequencies (Fig. 7D). Together, these data show that transcription of *trpE* is controlled through coordinated regulation of both burst size and frequency.

## DISCUSSION

Variability in mRNA levels arises from transcriptional bursting, an inherently stochastic process producing intermittent bursts of transcripts (*3*, *5*–*8*, *10*). These fluctuations are a major source of transcriptional noise in both eukaryotes and bacteria (*2*, *3*, *14*, *16*, *22*). Most work has focused on transcription initiation, where bursts are modulated by either frequency or burst size, depending on promoter architecture (*2*, *13*–*18*). Early models proposed uniform bursting in bacteria (*14*), but growing evidence shows that gene-specific features modulate bursting dynamics (*10*, *13*, *18*). In contrast, how postinitiation mechanisms such as premature termination of transcription via attenuation shape bursting remains poorly understood. Our results indicate that the regulatory context of transcription attenuation predicts which burst parameter is tuned, extending noise control beyond promoter-proximal events. Rather than acting merely as a downstream brake, attenuation plays an active role in the control of stochastic gene expression.

Here, we demonstrate that enhancer-independent transcription can also be regulated by burst frequency through the combined action of initiation and attenuation control. Such mechanisms broaden the landscape of bacterial strategies for shaping phenotypic heterogeneity, with direct relevance both for stress adaptation and for engineering of synthetic gene circuits that either minimize variability or exploit it to diversify population behavior.

We further show that at the native locus of biosynthetic pathways, transcriptional states in one subpopulation can influence others via metabolic cooperation, revealing a previously unrecognized source of heterogeneity through metabolite exchange. This diffusible feedback propagates transcriptional states between neighboring cells, amplifying variability beyond cell-intrinsic noise. Functionally, such cross-feeding not only reduces the metabolic burden and creates heterogeneity within the population but may also stabilize cooperative interactions and shape the overall community structure.

Comparing I + PI versus PI regulation, we observed the canonical inverse relationship between noise and mean expression associated with σ^70^-dependent genes and found that initiation control introduces additional cell-to-cell variability by adding a regulatory “decision point.” Dissection of the *trp* operon shows that in *B. subtilis*, TRAP/anti-TRAP–mediated attenuation is a key regulator of noise, whereas in *E. coli*, both TrpR and ribosome-mediated attenuation act as molecular switches. This reveals that even in promoters regulated at initiation, downstream attenuation can influence cell-to-cell variation. The differing regulatory architectures give rise to distinct patterns of transcriptional bursting, which may reflect contrasting evolutionary pressures and adaptive strategies.

In the PI system of *B. subtilis*, TRAP/anti-TRAP–mediated attenuation modulates burst size but not frequency. Anti-TRAP’s trimeric TRAP-binding state dampens attenuation, while its higher-order oligomer serves as a tunable reservoir that regulates the fraction of inhibitory anti-TRAP available for TRAP binding (*40*). Our data suggest that in WT, this organization generates controlled heterogeneity.

Anti-TRAP therefore provides a controlled protective buffer against TRAP-mediated overrepression that otherwise follows the rise in intracellular tryptophan. Without this buffering, high TRAP activity enforces strong transcriptional repression at medium and high levels of extracellular tryptophan. Cells lacking anti-TRAP (Δ*rtpA*) lose the graded transcriptional control, display a tail of cells with increased burst frequency in the presence of tryptophan, and exhibit increased transcriptional noise specifically at high tryptophan levels where buffering would normally be the strongest. Conversely, cells lacking TRAP (Δ*mtrB*) remove the controlled heterogeneity together and show uniformly high expression with low noise.

These architecture-dependent differences suggest that the *B. subtilis* system evolved not merely to implement attenuation but to preserve *trpE* expression in a subset of cells across a wide range of tryptophan concentrations. This could minimize the delay required to reinitiate tryptophan biosynthesis when local tryptophan levels abruptly collapse, ensuring rapid reacquisition of tryptophan production and, thus, biosynthetic capacity. Such a strategy may be particularly advantageous in spatially heterogeneous soil environments, where tryptophan availability fluctuates locally, driven by the heterogenous distribution of plant root exudates within the rhizosphere (*41*) and low tryptophan availability in bulk soil (*42*). Local tryptophan production by high-*trpE* subpopulations could also support neighboring cells and enable metabolic cooperation that stabilizes the community structure. The interaction between TRAP and anti-TRAP thus appears to act as an adaptive mechanism under spatially heterogeneous environments that preserves resilience without incurring excessive variability under nutrient-replete conditions.

In *E. coli*, the I + PI architecture combines TrpR-mediated initiation control with ribosome-mediated attenuation via *trpL*, yielding a two-layer regulation of burst parameters that suppresses transcription when tryptophan is present and permits rapid reactivation during scarcity. This may constitute an adaptation to the mammalian gut, where feeding cycles of the host repeatedly expose *E. coli* to alternating periods of feast and famine (*43*). Notably, it was recently shown that the gut microbiome obtains essential amino acids such as tryptophan from the host diet (*44*), subjecting the regulation of *trpE* transcription to temporal fluctuations in co-repressor (tryptophan) availability.

Mechanistically, our data suggest that at low tryptophan levels, the two regulatory layers act together to limit burst frequency and size, switching *trpE* transcription off. When tryptophan is absent, TrpR disengages and ribosomes stall while translating the leader peptide, releasing both constraints so *trpE* transcription is resumed. Initiation control therefore modulates the frequency and size of transcriptional bursts, while regulated attenuation alters the burst size in response to tryptophan. Removing TrpR (Δ*trpR*) uncouples transcription initiation from tryptophan, and hence, burst frequency remains high and tryptophan-insensitive. Attenuation alone produces a graded burst size reduction in these cells that is saturated at medium tryptophan levels. When attenuation is locked in its on-state while TrpR controls transcription initiation (Δ*trpL*), the productive output of initiated transcription is functionally overridden as nearly all transcripts terminate before *trpE*. Consequently, the frequency and size of *trpE* transcription bursts remain low regardless of external tryptophan levels and the associated rate of initiation. The higher *trpE* read-through in Δ*trpR*Δ*trpL* cells under all conditions suggests that premature termination can fail if initiation is uncontrolled. It further indicates that residual TrpR-dependent constraints on transcription initiation exist even in the absence of external tryptophan. Notably, *trpE* transcription in these cells remains sensitive to tryptophan, suggesting an additional TrpR- and *trpL*-independent layer of control, potentially exerted via small regulatory RNAs, as described previously (*39*).

Together, our study shows how transcriptional noise and bursting in bacteria arise from the interplay between transcription initiation and postinitiation control. We also show that metabolic cross-feeding can drive intercellular transcription regulation and alter cell-to-cell variation. Our findings further suggest that the regulatory architectures of genes evolve to match the dominant form of environmental uncertainty. Spatial heterogeneity in soil selects for controlled diversification in *B. subtilis*, while temporal fluctuation in the mammalian gut selects for rapid, coordinated regulation in *E. coli*. These results underscore the need to consider both molecular circuitry and environmental context when interpreting, predicting, and engineering microbial behavior.

## MATERIALS AND METHODS

### Bacterial strains

The *E. coli* BW25113 (WT) and Δ*trpR*, Δ*trpE*, and Δ*trpL* single-gene knockouts were acquired from the Keio Collection (all in the BW25113 background) (*45*). Mutant stains were rendered markerless by removing the kanamycin resistance cassette situated at the native locus of the target gene using Flp recombinase from the pCP20 plasmid. The Δ*trpR*Δ*trpL* double mutant was generated by P1 phage transduction using the Δ*trpL* strain as the donor and the Δ*trpR* strain as the recipient. *T*he Δ*trpE*, Δ*mtrB*, and Δ*rtpA* knockouts of *B. subtilis* were obtained from the *Bacillus* Genetic Stock Centre (BGSC) in a tryptophan auxotrophic (*trpC*2) background (*46*). Kanamycin markers of each mutant acquired were polymerase chain reaction amplified, gel purified, and transformed into tryptophan prototrophic WT (168 *trp*C+) using natural competence. Briefly, a single colony of donor *B. subtilis* tryptophan prototrophic WT (168 *trp*C+) strain was inoculated into SP medium at 37°C with shaking (150 rpm) overnight, then diluted 1:50 with 500 μl of fresh SP medium and 1 μl of purified polymerase chain reaction product, and incubated at 37°C with shaking (150 rpm) for 5.5 hours. Cultures were then centrifuged and resuspended in 1 ml of LB medium and incubated for 1.5 hours at 37°C with shaking (150 rpm). Cultures were then plated on LB medium supplemented with kanamycin (5 μg/ml). Kanamycin markers were excised using pDR244, a Cre recombinase–expressing plasmid, obtained from the BGSC (*46*). The Δ*mtrB* strain was transformed with the SG13 plasmid, containing a P*_veg_-gfp* transcriptional fusion, acquired from the BGSC (*46*) using natural competence. The resultant Δ*mtrB amyE::gfp* was used in the smRNA FISH coculture experiment.

### Growth assay

A single colony was inoculated into 5 ml of LB broth and incubated at 37°C, with shaking at 220 rpm. The next day, cell cultures were centrifuged at 5000 rpm for 5 min, and the supernatant was removed. The pellet was washed thrice in 1.5 ml of medium of interest (fresh M9 minimal media), each time centrifuging at 5000 rpm for 5 min. The optical density at 600 nm (OD_600_) was standardized to 0.1 in 500 μl. A volume of 100 μl of the experimental culture was transferred into a flat-bottom well of a 96-well microtiter plate, including three replicates of each condition. The OD_600_ was measured every hour for 40 hours using a FLUOstar Omega (BMG LABTECH) ultraviolet-visible filter–based microplate reader.

### smRNA FISH

Fluorescently labeled probes targeting the *trpE* mRNA transcripts were designed for both *E. coli* and *B. subtilis* organisms using Stellaris Probe Designer software (LGC Biosearch Technologies). Sequences of all fluorescently labeled probes used in this study are provided in table S3. Design parameters included an oligonucleotide length of 20 nucleotides, a minimum spacing of two nucleotides between probes, and a masking level set to 1or 2. All probes were conjugated with 6-carboxytetramethylrhodamine succinimidyl ester (6-TAMRA) as the fluorescent dye. A single colony of the target strain was picked using a sterile loop and transferred into 5 ml of LB broth in a sterile 30-ml polystyrene universal tube and incubated at 37°C, with shaking at 220 rpm. The next day, cell cultures were centrifuged at 5000 rpm for 5 min, and the supernatant was removed. The pellet was washed thrice in 1.5 ml of fresh modified M9 medium, each time centrifuging at 5000 rpm for 5 min. The OD_600_ was standardized to 0.1 in 20 ml of culture volume supplemented with 0, 5, 50, or 100 μM (corresponding to 0, 1×, 10×, and 20×) tryptophan and harvested at the midexponential phase (OD_600_ = 0.4) by centrifugation. The cells were resuspended and fixed in 1 ml of ice-cold 1× phosphate-buffered saline in diethyl pyrocarbonate (DEPC)–treated water containing 3.7% (v/v) formaldehyde and incubated for 30 min at room temperature. After fixation, the cells were washed twice with 1 ml of 1× phosphate-buffered saline in DEPC-treated water, then resuspended in 1 ml of 70% (v/v) ethanol in DEPC-treated water, and incubated for 1 hour at room temperature to permeabilize. Following permeabilization, the cells were washed with 1 ml of 2× SSC in DEPC-treated water containing 40% (w/v) formamide and incubated overnight at 30°C with hybridization buffer [2× SSC in DEPC-treated water, 40% (w/v) formamide, 10% (w/v) dextran sulfate, 2 mM ribonucleoside-vanadyl complex, and *E. coli* tRNA (1 mg/ml)] and 1 μM *trpE*-specific fluorescent probes. After hybridization, 10 μl of the cells was washed twice in 200 μl of ice-cold wash solution [40% (w/v) formamide and 2× SSC in DEPC-treated water] and incubated for 30 min at 30°C. The chromosomal DNA was then stained with 4′,6-diamidino-2-phenylindole (DAPI)–containing wash solution [40% (w/v) formamide, 2× SSC in DEPC-treated water, and DAPI (10 μg/ml)] for 30 min at 30°C. The cells were resuspended in 100 μl of 2× SSC in DEPC-treated water, from which 2 μl was spotted onto the center of a 1% (w/v) agarose gel. Once dry, a 1- by 1-cm square was cut around the sample and transferred to a 76- by 26-mm microscope glass slide.

### Quantification of *trpE* mRNA molecules

Cells were imaged using a Leica Stellaris 8 confocal microscope, acquiring five *z*-slices at 200-nm intervals for each channel (bright field, DAPI, and 6-TAMRA) across multiple *x*/*y* (stage) positions. The resulting 16-bit .tif images from all three channels were used to generate cell segmentations using Schnitzcells software (*47*) in MATLAB (MathWorks). Two representative, unprocessed raw smRNA FISH image sets for every strain and each tryptophan concentration tested are deposited at Zenodo (DOI: 10.5281/zenodo.18632896). Images are provided for the bright-field, DAPI, and TAMRA channels. All segmentations were visually inspected and manually corrected where necessary; cells with out-of-focus or poorly resolved TAMRA signal were excluded from downstream analysis. The *trpE* mRNA copy numbers in single cells were quantified with the Spätzcells program (*36*) in MATLAB using the 6-TAMRA channel images and the cell segmentations. Fluorescent spots within the segmented cells were detected and differentiated from nonspecific background signals by setting a false-positive threshold using Δ*trpE* cells as a negative control. False-positive spots were excluded by setting the threshold at the 99.9th percentile of spot intensities observed in Δ*trpE* cells. Fluorescent spots exceeding the false-positive threshold were classified as specific signals corresponding to *trpE* mRNA molecules hybridized with complementary DNA probes. These fluorescent spots’ peak height and intensity were analyzed to determine the mRNA copy numbers. The intensity distribution of spots from a low-expressing control strain was fitted to a multi-Gaussian function, with the mean of the first Gaussian representing the intensity of a single mRNA molecule. The total fluorescence intensity of spots in each cell was divided by the intensity of a single mRNA molecule to calculate the number of mRNA molecules per cell. These data were then used to calculate the relative frequencies, mean, and standard deviation of mRNA copy numbers across the population.

### Coculture smRNA FISH

Single colonies of WT and Δ*mtrB* were inoculated in 5 ml of LB broth in separate tubes and incubated at 37°C, with shaking at 220 rpm overnight. The cultures were then centrifuged at 5000 rpm for 5 min, and the supernatant was removed. The pellet was washed thrice in 1.5 ml of modified M9 medium, each time centrifuging at 5000 rpm for 5 min. The OD_600_ of each strain was standardized to 0.05 in a 20 ml of culture volume of fresh modified M9 medium incubated at 37°C, with shaking at 220 rpm, and harvested at the midexponential phase (OD_600_ = 0.4) by centrifugation. Once harvested, the cells were treated for smRNA FISH as previously described (*36*). For the coculture experiment, along with bright-field, DAPI, and 6-TAMRA channel images, GFP images were also captured to distinguish the GFP–fluorescently tagged Δ*mtrB* cells within the images such that only cell segmentation of non–GFP-fluorescent WT cells was generated using Schnitzcells software (*47*) in MATLAB (MathWorks).

### Amino acid measurements

Single colonies of *E. coli and B. subtilis* WT strains (BW25113 and 168 *trpC+*, respectively) were grown overnight in 5 ml of LB broth at 37°C, with shaking at 220 rpm. The next day, the cultures were centrifuged at 5000 rpm for 5 min, and the supernatant was discarded. The pellets were then resuspended and washed in M9 minimal medium thrice, each time centrifuging at 5000 rpm for 5 min. The OD_600_ was adjusted to 0.1 in 15 ml of M9 minimal media incubated at 37°C, with shaking at 220 rpm for 24 hours. One milliliter of the culture was filter sterilized with Millipore 0.2-μm filter to remove the cells. The spent media were then immediately frozen in liquid nitrogen and stored at −80°C. The amino acids from the spent medium were derivatized using the AccQ-Tag kit (Waters) as per the manufacturer’s guidelines and quantified by tandem mass spectrometry using a TQSμ coupled to a Acquity UPLC equipped with an HSS T3 2.1- by 150-mm, 1.8-μm column. Separation was achieved using a gradient with phase A water with 0.1% formic acid (v/v) and acetonitrile with 0.1% formic acid (B) and the column held at 45°C. Gradient elution was performed with 4% B at 0.6 ml/min starting and held for 0.5 min, then to 10% B over 2 min, then to 28% B over 2.5 min, and to 95% B for 1 min before returning to 4% B (1.3 min) for re-equilibration. The amino acid derivatives were quantified in positive mode, as published previously (*48*). Data were analyzed using an in-house MATLAB pipeline based on software published by Behrends *et al.* (*49*). Values are expressed as arbitrary units (A/U).

### Computational analysis of burst kinetics

Burst size, frequency, and transcriptional repression were estimated using a Bayesian inference framework, as described in (*13*). A zero-inflated negative binomial model was used to describe the distribution of mRNA copy numbers. Parameters θ = [ω,*r*,*p*] were inferred using Metropolis-Hastings Markov chain Monte Carlo (MCMC) sampling, with the posterior distribution being computed by the product of the likelihood and the priors. Uniform priors were used for ω and *p* (0,1), while *r* was assigned from a half-normal (μ = 0, σ = 20) positively truncated prior. Sampling was performed using a custom MCMC implementation with a multivariate Gaussian proposal distribution, in which the standard deviation was set to 5% of the current parameter values to ensure good convergence. Chains were iterated for 500,000 steps, with 100,000 discarded as burn-in and thinning applied by a factor of 100. Burst parameters, maximum a posteriori estimate (measure of the center of the error bar), and 95% credible intervals were computed from the posterior distributions for further interpretation.

## References

[1] A. Sanchez, I. Golding, Genetic determinants and cellular constraints in noisy gene expression. Science 342, 1188–1193 (2013). 24311680 10.1126/science.1242975 PMC4045091

[2] I. Golding, J. Paulsson, S. M. Zawilski, E. C. Cox, Real-time kinetics of gene activity in individual bacteria. Cell 123, 1025–1036 (2005). 16360033 10.1016/j.cell.2005.09.031

[3] A. J. M. Larsson, P. Johnsson, M. Hagemann-Jensen, L. Hartmanis, O. R. Faridani, B. Reinius, A. Segerstolpe, C. M. Rivera, R. Bing, R. Sandberg, Genomic encoding of transcriptional burst kinetics. Nature 565, 251–254 (2019). 30602787 10.1038/s41586-018-0836-1 PMC7610481

[4] M. M. K. Hansen, R. V. Desai, M. L. Simpson, L. S. Weinberger, Cytoplasmic amplification of transcriptional noise generates substantial cell-to-cell variability. Cell Syst. 7, 384–397.e6 (2018). 30243562 10.1016/j.cels.2018.08.002 PMC6202163

[5] J. Rodriguez, D. R. Larson, Transcription in living cells: Molecular mechanisms of bursting. Annu. Rev. Biochem. 89, 189–212 (2020). 32208766 10.1146/annurev-biochem-011520-105250

[6] D. Hebenstreit, P. Karmakar, Transcriptional bursting: From fundamentals to novel insights. Biochem. Soc. Trans. 52, 1695–1702 (2024). 39119657 10.1042/BST20231286 PMC11668302

[7] E. A. Leyes Porello, R. T. Trudeau, B. Lim, Transcriptional bursting: Stochasticity in deterministic development. Development 150, dev201546 (2023). 37337971 10.1242/dev.201546 PMC10323239

[8] E. Tunnacliffe, J. R. Chubb, What is a transcriptional burst? Trends Genet. 36, 288–297 (2020). 32035656 10.1016/j.tig.2020.01.003

[9] M. B. Elowitz, A. J. Levine, E. D. Siggia, P. S. Swain, Stochastic gene expression in a single cell. Science 297, 1183–1186 (2002). 12183631 10.1126/science.1070919

[10] D. Jones, J. Elf, Bursting onto the scene? Exploring stochastic mRNA production in bacteria. Curr. Opin. Microbiol. 45, 124–130 (2018). 29705632 10.1016/j.mib.2018.04.001

[11] M. Ackermann, A functional perspective on phenotypic heterogeneity in microorganisms. Nat. Rev. Microbiol. 13, 497–508 (2015). 26145732 10.1038/nrmicro3491

[12] A. J. Grimbergen, J. Siebring, A. Solopova, O. P. Kuipers, Microbial bet-hedging: The power of being different. Curr. Opin. Microbiol. 25, 67–72 (2015). 26025019 10.1016/j.mib.2015.04.008

[13] C. Engl, G. Jovanovic, R. D. Brackston, I. Kotta-Loizou, M. Buck, The route to transcription initiation determines the mode of transcriptional bursting in *E. coli*. Nat. Commun. 11, 2422 (2020). 32415118 10.1038/s41467-020-16367-6 PMC7229158

[14] LH. So, A. Ghosh, C. Zong, L. A. Sepúlveda, R. Segev, I. Golding, General properties of transcriptional time series in *Escherichia coli*. Nat. Genet. 43, 554–560 (2011). 21532574 10.1038/ng.821 PMC3102781

[15] K. Fujita, M. Iwaki, T. Yanagida, Transcriptional bursting is intrinsically caused by interplay between RNA polymerases on DNA. Nat. Commun. 7, 13788 (2016). 27924870 10.1038/ncomms13788 PMC5151093

[16] R. D. Dar, B. S. Razooky, A. Singh, T. V. Trimeloni, J. M. McCollum, C. D. Cox, M. L. Simpson, L. S. Weinberger, Transcriptional burst frequency and burst size are equally modulated across the human genome. Proc. Natl. Acad. Sci. U.S.A. 109, 17454–17459 (2012). 23064634 10.1073/pnas.1213530109 PMC3491463

[17] E. Tunnacliffe, A. M. Corrigan, J. R. Chubb, Promoter-mediated diversification of transcriptional bursting dynamics following gene duplication. Proc. Natl. Acad. Sci. U.S.A. 115, 8364–8369 (2018). 30061408 10.1073/pnas.1800943115 PMC6099902

[18] S. Chong, C. Chen, H. Ge, X. S. Xie, Mechanism of transcriptional bursting in bacteria. Cell 158, 314–326 (2014). 25036631 10.1016/j.cell.2014.05.038 PMC4105854

[19] A. Klindziuk, B. Meadowcroft, A. B. Kolomeisky, A mechanochemical model of transcriptional bursting. Biophys. J. 118, 1213–1220 (2020). 32049059 10.1016/j.bpj.2020.01.017 PMC7063482

[20] H. P. Patel, S. Coppola, W. Pomp, U. Aiello, I. Brouwer, D. Libri, T. L. Lenstra, DNA supercoiling restricts the transcriptional bursting of neighboring eukaryotic genes. Mol. Cell 83, 1573–1587.e8 (2023). 37207624 10.1016/j.molcel.2023.04.015 PMC10205079

[21] C. L. Turnbough, Regulation of bacterial gene expression by transcription attenuation. Microbiol. Mol. Biol. Rev. 83, e00019 (2019). 31270135 10.1128/MMBR.00019-19 PMC6710462

[22] J. M. Raser, E. K. O’Shea, Noise in gene expression: Origins, consequences, and control. Science 309, 2010–2013 (2005). 16179466 10.1126/science.1105891 PMC1360161

[23] P. Babitzke, Regulation of tryptophan biosynthesis: Trp-ing the TRAP or how *Bacillus subtilis* reinvented the wheel. Mol. Microbiol. 26, 1–9 (1997). 9383185 10.1046/j.1365-2958.1997.5541915.x

[24] C. Yanofsky, K. V. Konan, J. P. Sarsero, Some novel transcription attenuation mechanisms used by bacteria. Biochimie 78, 1017–1024 (1996). 9150880 10.1016/s0300-9084(97)86725-9

[25] P. Gollnick, P. Babitzke, A. Antson, C. Yanofsky, Complexity in regulation of tryptophan biosynthesis in *Bacillus subtilis*. Annu. Rev. Genet. 39, 47–68 (2005). 16285852 10.1146/annurev.genet.39.073003.093745

[26] H. Shimotsu, M. I. Kuroda, C. Yanofsky, D. J. Henner, Novel form of transcription attenuation regulates expression the *Bacillus subtilis* tryptophan operon. J. Bacteriol. 166, 461–471 (1986). 2422155 10.1128/jb.166.2.461-471.1986 PMC214627

[27] T. Platt, Termination of transcription and its regulation in the tryptophan operon of *E. coli*. Cell 24, 10–23 (1981). 7016334 10.1016/0092-8674(81)90496-7

[28] C. Yanofsky, R. L. Kelley, V. Horn, Repression is relieved before attenuation in the trp operon of *Escherichia coli* as tryptophan starvation becomes increasingly severe. J. Bacteriol. 158, 1018–1024 (1984). 6233264 10.1128/jb.158.3.1018-1024.1984 PMC215544

[29] C. Yanofsky, The different roles of tryptophan transfer RNA in regulating trp operon expression in *E. coli* versus *B. subtilis*. Trends Genet. 20, 367–374 (2004). 15262409 10.1016/j.tig.2004.06.007

[30] N. M. McAdams, P. Gollnick, Characterization of TRAP-mediated regulation of the *B. subtilis* trp operon using in vitro transcription and transcriptional reporter fusions in vivo. Methods Mol. Biol. 1259, 333–347 (2015). 25579595 10.1007/978-1-4939-2214-7_20

[31] A. Valbuzzi, P. Gollnick, P. Babitzke, C. Yanofsky, The anti-trp RNA-binding attenuation protein (Anti-TRAP), AT, recognizes the tryptophan-activated RNA binding domain of the TRAP regulatory protein. J. Biol. Chem. 277, 10608–10613 (2002). 11786553 10.1074/jbc.M111813200

[32] P. Babitzke, P. Gollnick, Posttranscription initiation control of tryptophan metabolism in *Bacillus subtilis* by the *trp* RNA-binding attenuation protein (TRAP), anti-TRAP, and RNA structure. J. Bacteriol. 183, 5795–5802 (2001). 11566976 10.1128/JB.183.20.5795-5802.2001 PMC99655

[33] N. M. McAdams, P. Gollnick, The *Bacillus subtilis* TRAP protein can induce transcription termination in the leader region of the tryptophan biosynthetic (trp) operon independent of the trp attenuator RNA. PLOS ONE 9, e88097 (2014). 24505391 10.1371/journal.pone.0088097 PMC3913778

[34] C. L. Squires, F. D. Lee, C. Yanofsky, Interaction of the trp repressor and RNA polymerase with the trp operon. J. Mol. Biol. 92, 93–111 (1975). 1097702 10.1016/0022-2836(75)90093-5

[35] G. Bogosian, R. L. Somerville, Analysis in vivo of factors affecting the control of transcription initiation at promoters containing target sites for Trp repressor. Mol. Gen. Genet. 193, 110–118 (1984). 6318045 10.1007/BF00327423

[36] S. O. Skinner, L. A. Sepúlveda, H. Xu, I. Golding, Measuring mRNA copy number in individual *Escherichia coli* cells using single-molecule fluorescent in situ hybridization. Nat. Protoc. 8, 1100–1113 (2013). 23680982 10.1038/nprot.2013.066 PMC4029592

[37] L. R. Cruz-Vera, M. Gong, C. Yanofsky, Physiological effects of anti-TRAP protein activity and tRNA ^Trp^ charging on *trp* operon expression in *Bacillus subtilis*. J. Bacteriol. 190, 1937–1945 (2008). 18178730 10.1128/JB.01820-07 PMC2258860

[38] W. J. Yang, C. Yanofsky, Effects of tryptophan starvation on levels of the *trp* RNA-binding attenuation protein (TRAP) and anti-TRAP regulatory protein and their influence on *trp* operon expression in *Bacillus subtilis*. J. Bacteriol. 187, 1884–1891 (2005). 15743934 10.1128/JB.187.6.1884-1891.2005 PMC1064063

[39] J. McQuail, G. Matera, T. Gräfenhan, T. Bischler, P. Haberkant, F. Stein, J. Vogel, S. Wigneshweraraj, Global Hfq-mediated RNA interactome of nitrogen starved *Escherichia coli* uncovers a conserved post-transcriptional regulatory axis required for optimal growth recovery. Nucleic Acids Res. 52, 2323–2339 (2024). 38142457 10.1093/nar/gkad1211 PMC10954441

[40] C. A. McElroy, E. C. Ihms, D. K. Yadav, M. L. Holmquist, V. Wadhwa, V. H. Wysocki, P. Gollnick, M. P. Foster, Solution structure, dynamics and tetrahedral assembly of Anti-TRAP, a homo-trimeric triskelion-shaped regulator of tryptophan biosynthesis in *Bacillus subtilis*. J. Struct. Biol. X 10, 100103 (2024). 39035014 10.1016/j.yjsbx.2024.100103 PMC11255114

[41] C. H. Jaeger III, S. E. Lindow, W. Miller, E. Clarke, M. K. Firestone, Mapping of sugar and amino acid availability in soil around roots with bacterial sensors of sucrose and tryptophan. Appl. Environ. Microbiol. 65, 2685–2690 (1999). 10347061 10.1128/aem.65.6.2685-2690.1999 PMC91396

[42] M. Lebhuhn, B. Heilmann, A. Hartmann, Effects of drying/rewetting stress on microbial auxin production and L-tryptophan catabolism in soils. Biol. Fert. Soils 18, 302–310 (1994).

[43] S. Doranga, K. A. Krogfelt, P. S. Cohen, T. Conway, Nutrition of *Escherichia coli* within the intestinal microbiome. EcoSal Plus 12, eesp00062023 (2024). 38417452 10.1128/ecosalplus.esp-0006-2023 PMC11636361

[44] X. Zeng, X. Xing, M. Gupta, F. C. Keber, J. G. Lopez, Y. J. Lee, A. Roichman, L. Wang, M. D. Neinast, M. S. Donia, M. Wuhr, C. Jang, J. D. Rabinowitz, Gut bacterial nutrient preferences quantified in vivo. Cell 185, 3441–3456.e19 (2022). 36055202 10.1016/j.cell.2022.07.020 PMC9450212

[45] T. Baba, T. Ara, M. Hasegawa, Y. Takai, Y. Okumura, M. Baba, K. A. Datsenko, M. Tomita, B. L. Wanner, H. Mori, Construction of *Escherichia coli* K-12 in-frame, single-gene knockout mutants: The Keio collection. Mol. Syst. Biol. 2, 2006.0008 (2006). 10.1038/msb4100050 PMC1681482 16738554

[46] B. M. Koo, G. Kritikos, J. D. Farelli, H. Todor, K. Tong, H. Kimsey, I. Wapinski, M. Galardini, A. Cabal, J. M. Peters, A.-B. Hachmann, D. Z. Rudner, K. N. Allen, A. Typas, C. A. Gross, Construction and analysis of two genome-scale deletion libraries for *Bacillus subtilis*. Cell Syst. 4, 291–305.e7 (2017). 28189581 10.1016/j.cels.2016.12.013 PMC5400513

[47] J. W. Young, C. W. Locke, A. Altinok, N. Rosenfeld, T. Bacarian, P. S. Swain, E. Mjolsness, M. B. Elowitz, Measuring single-cell gene expression dynamics in bacteria using fluorescence time-lapse microscopy. Nat. Protoc. 7, 80–88 (2012). 10.1038/nprot.2011.432 PMC4161363 22179594

[48] N. Gray, R. Zia, A. King, V. C. Patel, J. Wendon, M. J. W. McPhail, M. Coen, R. S. Plumb, I. D. Wilson, J. K. Nicholson, High-speed quantitative UPLC-MS analysis of multiple amines in human plasma and serum via precolumn derivatization with 6-aminoquinolyl-*N*-hydroxysuccinimidyl carbamate: Application to acetaminophen-induced liver failure. Anal. Chem. 89, 2478–2487 (2017). 28194962 10.1021/acs.analchem.6b04623

[49] V. Behrends, G. D. Tredwell, J. G. Bundy, A software complement to AMDIS for processing GC-MS metabolomic data. Anal. Biochem. 415, 206–208 (2011). 21575589 10.1016/j.ab.2011.04.009

