# Supplementary material for: Premature transcription termination modulates stochastic gene expression in bacteria
Source: Sci Adv. 2026 May 15;12(20):eaed0831. doi: 10.1126/sciadv.aed0831 (PMC13178558; doi:10.1126/sciadv.aed0831)
Supplement: Supplementary file 1 — Figs. S1 to S7 Tables S1 to S3 Legends for data S1 to S5 [file sciadv.aed0831_sm.pdf]

Science Advances
AAAS

Supplementary Materials for

Premature transcription termination modulates stochastic gene expression in bacteria

Shafagh Moradian *et al.*

Corresponding author: Christoph Engl, c.engl@qmul.ac.uk

Sci. Adv. **12**, eaed0831 (2026)
DOI: 10.1126/sciadv.aed0831

The PDF file includes:

Figs. S1 to S7
Tables S1 to S3
Legends for data S1 to S5

Other Supplementary Material for this manuscript includes the following:

Data S1 to S5

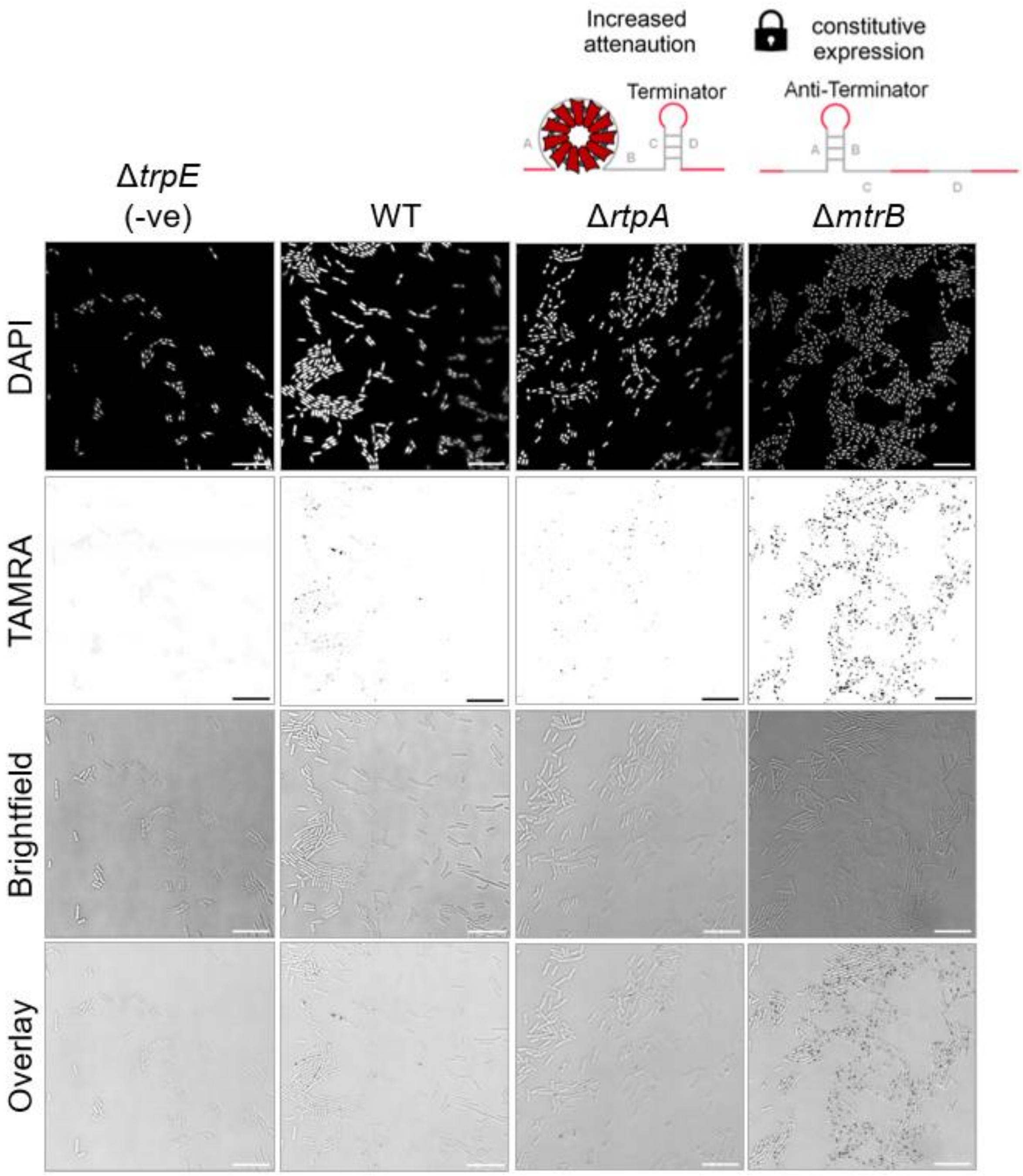

Fig. S1. Representative smFISH images targeting *trpE* in *B. subtilis*. Shown are example images of DAPI, TAMRA, brightfield, and TAMRA + brightfield overlays for *B. subtilis*. The Δ*trpE* negative control (-ve) display**s** an absence of TAMRA signal. WT cells show low TAMRA signal**,** while the Δ*mtrB* mutant exhibits a markedly increased TAMRA signal relative to the WT. The size of the scale bar is 10 μm.

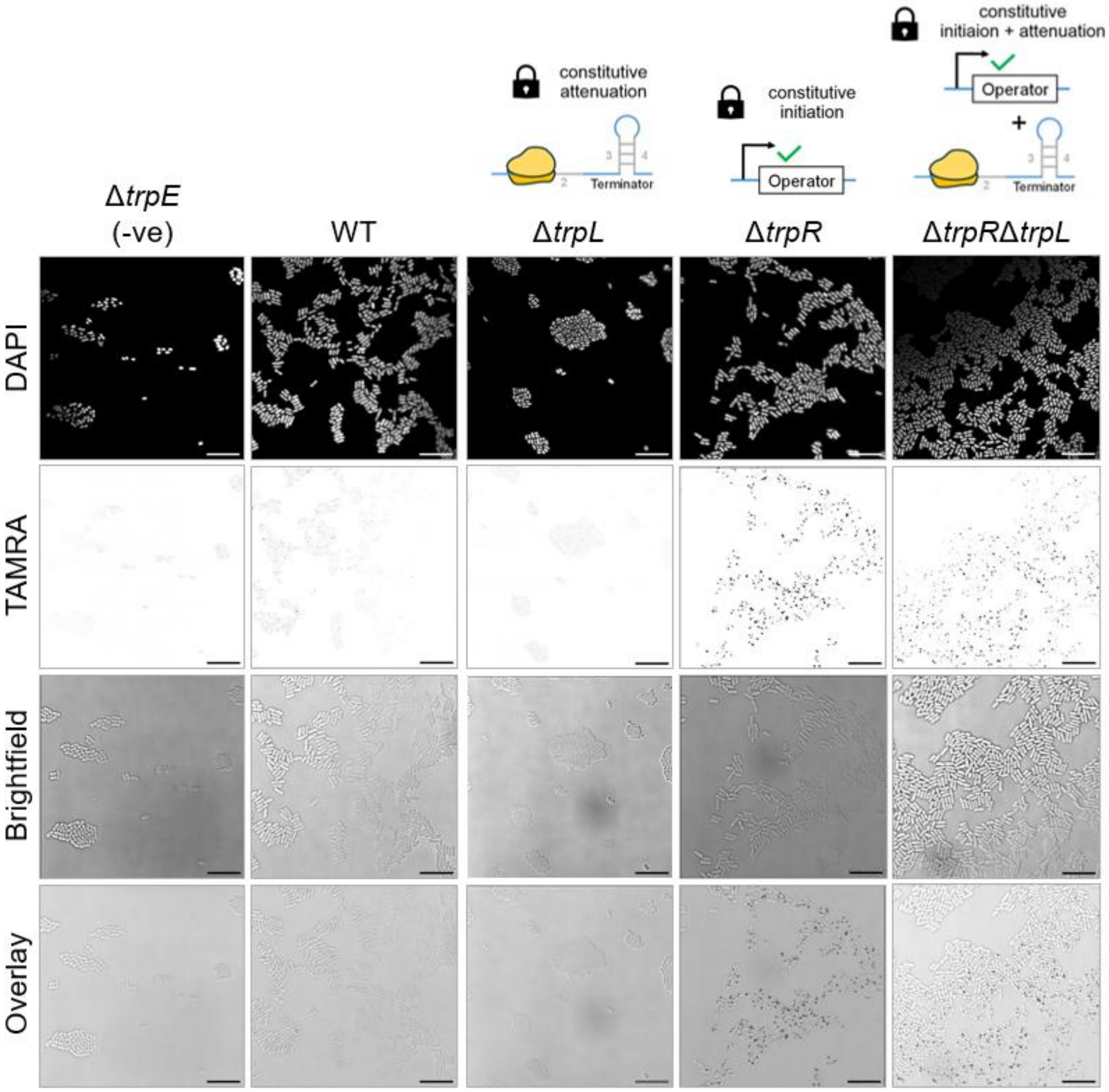

Fig. S2. Representative smFISH images targeting *trpE* in *E. coli*. Shown are example images of DAPI, TAMRA, brightfield, and TAMRA + brightfield overlays for *E. coli*. The Δ*trpE* negative control (-ve) displays an absence of TAMRA signal. WT cells show low TAMRA signal while the Δ*trpR* mutant shows increased TAMRA signal compared to the WT. The size of the scale bar is 10 μm.

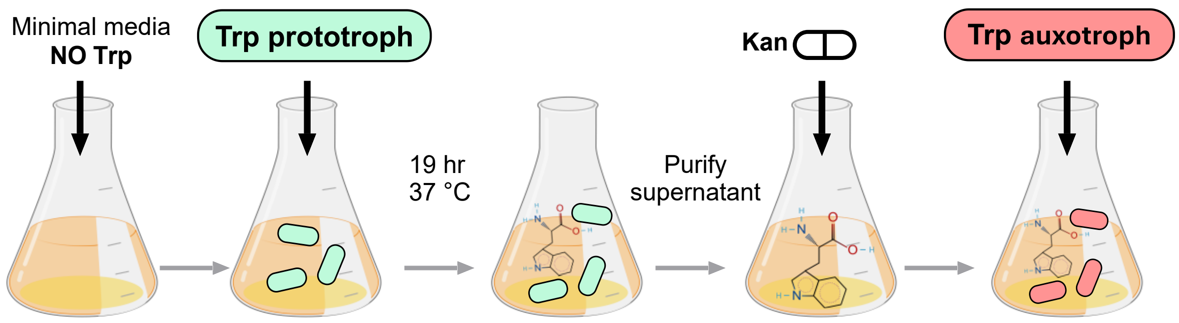

Fig. S3. Experimental set up using tryptophan auxotrophs as a biosensor. Tryptophan prototrophs were cultured in minimal media lacking tryptophan. Following the purification of the supernatant after a 19-hour incubation, kanamycin was added and the tryptophan auxotroph strain (Δ*trpE*::*kan*) was cultured and growth was monitored. Growth data is presented in supplementary Fig. S4.

Amino acid	*B. subtilis*	*E. coli*
**Glycine**	Present	Present
**Alanine**	Present	Present
**Leu/Ile**	Present	Present
**Glutamate**	Present	Present
**Phenylalanine**	Present	Present
**Tyrosine**	Present	Present
**Tryptophan**	Present	Present
**Lysine**	Absent	Absent
**Asparagine**	Absent	Absent
**Aspartate**	Absent	Absent
**Methionine**	Absent	Absent
**Histidine**	Absent	Absent
**Arginine**	Absent	Absent
**Citrulline**	Absent	Absent

Table S1. Amino acids identified either as present or absent in the supernatant of WT cells at 24 hours post inoculation using mass spectrometry.

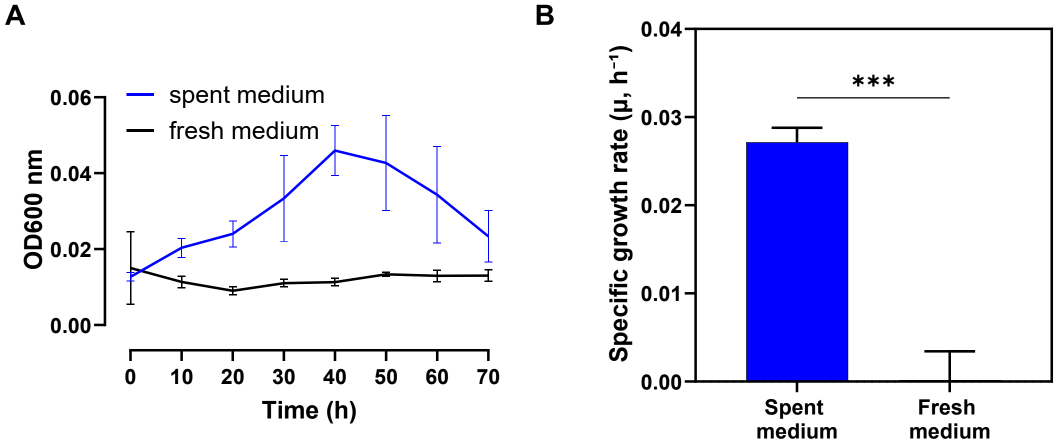

Fig. S4. Tryptophan cross feeding between tryptophan prototrophic doners and auxotrophic recipients. (A) Growth curve of a *B. subtilis* tryptophan auxotroph strain (Δ*trpE*) cultured in either fresh minimal medium or spent medium collected from tryptophan prototrophic WT. Bacterial growth was monitored over 10 hours over 70-hour OD600 using an Omega microplate reader in 100 μL culture volumes. Data represent the mean ± standard deviation from three independent experiments. (B) Specific growth rate (μ, h^{-1}) of the tryptophan auxotroph cultured in spent versus fresh medium, calculated from OD600 values between 10 and 40 hours using the exponential growth rate formula (μ = ln(ODt2)−ln(ODt1) / t2−t1). Bars represent the mean ± standard deviation of three biological replicates. Statistical significance was determined using an unpaired *t*-test (p = 0.0002) (GraphPad Software Inc.).

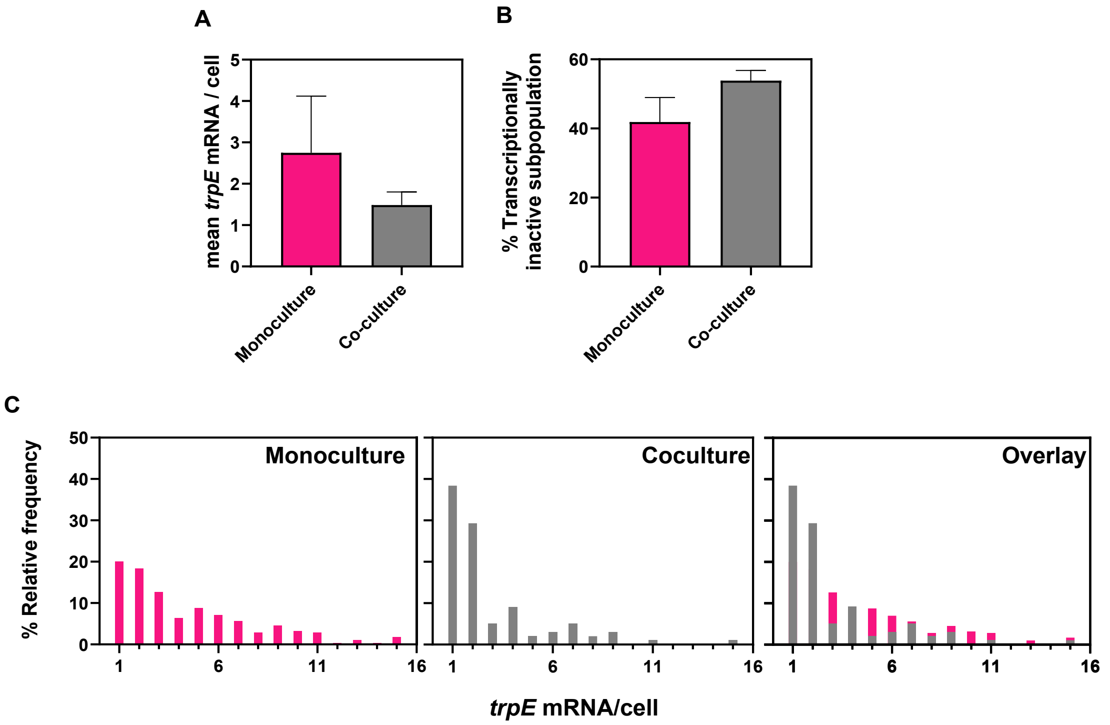

Fig. S5. Co-culture with high *trpE*-expressing mutant suppresses *trpE* transcription dynamics in wildtype cells. Expression dynamics of *trpE* in WT cells grown either as a monoculture or in co-culture with a high *trpE*-expressing TRAP mutant (Δ*mtrB*). Co-cultures were established at a 1:1 ratio of WT and Δ*mtrB* cells in minimal medium without supplemented tryptophan, grown to mid-exponential phase, and fixed with formaldehyde prior to hybridisation with TAMRA-labelled DNA probes targeting *trpE* mRNA. Cells were imaged using a Leica Stellaris 8 confocal microscope. Using Schnitzcells cell segmentation software (47), WT cells lacking GFP fluorescence were selected for quantification of *trpE* mRNA. (A) The mean number of *trpE* mRNA molecules per WT cell, quantified using smRNA FISH (36), in the WT monoculture and WT + Δ*mtrB* co-culture condition. (B) The proportion of WT cells in a transcriptionally inactive state (defined as having <1 *trpE* mRNA molecule), in the WT monoculture and WT + Δ*mtrB* co-culture condition. (C) Percentage relative frequency of *trpE* mRNA copy numbers in transcriptionally active (defined as cells expressing 1 or more molecules of *trpE* mRNA) WT cells in the WT monoculture and WT + Δ*mtrB* co-culture condition.

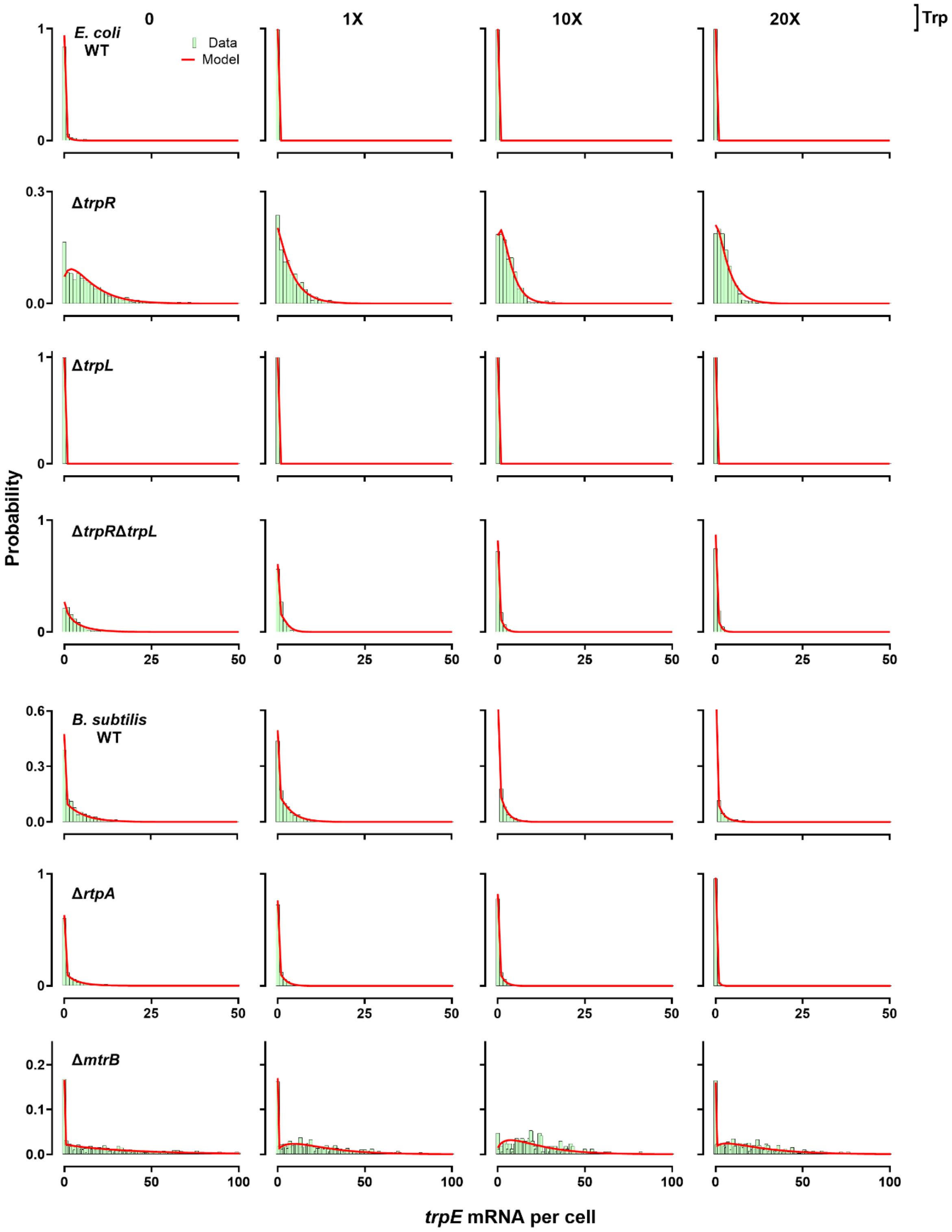

Fig. S6. Measured and ZINB-modelled mRNA distributions. Measured single-cell mRNA probability distributions of all strains and conditions in green, overlaid with predictions from the fitted zero-inflated negative binomial (ZINB) model.

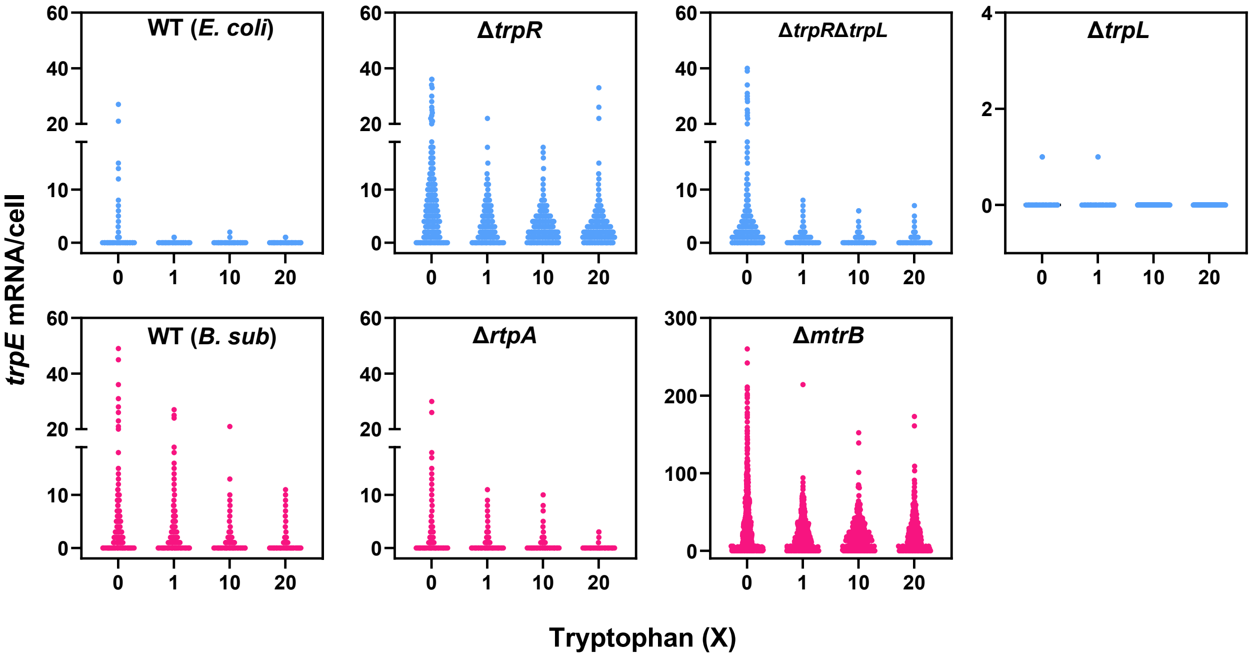

Fig. S7. Single molecules of *trpE* mRNA quantified in single cells of *B. subtilis* and *E. coli* strains in response to environmental tryptophan. Single molecules of *trpE* mRNA were quantified using Spätzcells software (36), and the resulting mRNA distributions are shown as scatterplots. The top panel (blue) represents all *E. coli* strains analysed in this study, while the bottom panel (pink) shows all *B. subtilis* strains.

E. coli				
	**WT**			
**Tryptophan (X)**	**0**	**1**	**10**	**20**
**Mean**	0.623	0.015	0.011	0.006
**SD**	0.152	0.009	0.008	0.003
	**Δ*trpR***			
**Tryptophan (X)**	**0**	**1**	**10**	**20**
**Mean**	5.298	2.654	2.345	2.710
**SD**	2.173	2.014	0.856	0.629
	**Δ*trpL***			
**Tryptophan (X)**	**0**	**1**	**10**	**20**
**Mean**	0.006	0.002	0	0
**SD**	0.002	0.00009	0	0
	**Δ*trpR* Δ*trpL***			
**Tryptophan (X)**	**0**	**1**	**10**	**20**
**Mean**	3.823	0.731	0.460	0.378
**SD**	1.431	0.045	0.024	0.122
B. subtilis				
	**WT**			
**Tryptophan (X)**	**0**	**1**	**10**	**20**
**Mean**	2.731	1.632	0.758	0.418
**SD**	0.971	0.831	0.256	0.249
	**Δ*rtpA***			
**Tryptophan (X)**	**0**	**1**	**10**	**20**
**Mean**	1.663	0.750	0.487	0.059
**SD**	0.029	0.131	0.087	0.003
	**Δ*mtrB***			
**Tryptophan (X)**	**0**	**1**	**10**	**20**
**Mean**	35.214	21.814	21.411	20.495
**SD**	6.568	1.443	1.795	3.631

Table S2. The mean and standard deviation of *trpE* mRNA levels quantified in every condition and strain tested in this study using smRNA FISH.

A	*E. coli*	
tttgtgtttttggctgagag	ccttttattgacgggactaa	caagtgacctgtctctagag
gaatagcgctgttagggtgg	aatagagcgactttgcgact	cggcataacttgacctttac
aaaagtggtcaacacacccc	actggtagtctttttttcgt	atggctagtatttctcgaca
cttaggcgtctatagctgtc	cataagtccggtcggacaaa	cttgtagactacgaccaact
actaaatttttcggacgacg	ctttttgttgcagagtgacg	cattactagaccgtgcgtaa
cgacgcgtaatgtcgaaatc	gacttgcttgatgcagtcgt	ggctagagtggtttcaactg
cagtgttaggtccgtgaaag	ggcgtatacgcaacacttac	aaggatacactacgtggagc
gaggaccgtgatgacctatt	tagtctcgctacttctcaag	cttgacgcagtgctagaact
tcacttgttagtggtttgac	acgcaaacaacgtttttcgc	atacttatacccctgcaatt
gacagtcaggtgacgaccta	cgacctctttaaaaggtcca	gcgatacgtcaattaacggc
gcgaatacgagggaaagcca	tagagcggcaaagagagacg	catccaataaagtggcgcgt
actgcgaaaggcaaataacg	ccggataatgcacgactttt	ctagagctgtggacgtaaca
gacaacttacatggcttcct	agggtcgggcatgtacaaaa	acgaccacatcaggaactaa
tgctcttcggtacaagaagc	taaagtgggataaaccgcgc	gctgctttgggcattgtttc
ccggacaagagaatactgga	ctttcgagcgagttcatact	gcgacatgacgcgcgataac
cgccctaaacttctaaatgg	ggtctaactctagatgggct	gtagtacgtgtcctctgaaa

B	*B. subtilis*	
ggcgtaaaaatctcctgtcg	cggcctgtaattaacgcata	gcctcgtaatgtacgagcaa
gctaacacctctggaagtgt	tactttggtttttgcaggtg	agaacgggctttgctatagc
gctatgtgactgtgggtaag	ataggttatacgttccgagt	tcatcgtctcataccaagac
tactatctcttcgaactgtc	tctcctttgtttttgcttt	cggcctcaagtgtttttaac
agaagaactttcgttcctga	ggtagttttagtagacctcg	acgtgtaatagagccaccaa
tgtaggtgaaccaggtctat	tttactacctggtttttttg	gctaactttttccccaagt
gcaaatagccggacttaggt	tatgttctgtgggtcgaaac	gacagctacgtgactacaga
gtgttaatttcttctcgtcc	tagccgaaaataccgactac	cgtcgaaaacgttcttgagc
caaaaagccggcgactagtc	attttcgtccgctatagaag	ctcccacataacggatgtaa
tgtcctttacttgattttct	gtgtttttaaactccacggc	caaactgcccttatagctga
cttgacctacttatggtgta	tcaatatggctcacgaatcc	gctaagcgtgctactcacat
ttttagttttgtggactcga	acgatctgtctctttatcag	ttgccacaacgtagctatgt
gtaaggaaaacagccgcctc	gccttgccaattatgtgcaa	cgaccgtaacaacgactaag
agcccatgaattcgatacta	atctttaggtaggctaacgg	ttctttcgacattatttcgg
ctcggaagacaaggaagcgt	gacttctactctctgacttc	gcgacgacttttgctaagta
ttgtctgtaccttttcacat	cgagtacttcctactttttc	aagtatcgttcctcctattt

Table S3. Sequences of fluorescently labelled probes targeting *trpE* mRNA in (A) *E. coli* and (B) *B. subtilis*. Probes were designed using the Stellaris Probe Designer (LGC Biosearch Technologies) with an oligonucleotide length of 20 nucleotides, a minimum spacing of 2 nucleotides, and a masking level of 1–2.

Legends for data S1 to S5

Data S1. Copy number of *trpE* mRNAs per cell of *Bacillus subtilis* wildtype, *ΔrtpA* and *ΔmtrB* mutant in presence and absence of external tryptophan.

Data S2. Copy number of *trpE* mRNAs per cell of *Escherichia coli* wildtype, *ΔtrpR, ΔtrpL*, and *ΔtrpRL* mutant in presence and absence of external tryptophan.

Data S3. Copy number of *trpE* mRNAs per cell of *Bacillus subtilis* wildtype in co-culture with high *trpE*-expressing TRAP mutant (*ΔmtrB*) in absence of external tryptophan.

Data S4. MCMC output after computational analysis of transcriptional burst kinetics.

Data S5. MAP estimates after computational analysis of transcriptional burst kinetics.
